# Detecting the QTL-Allele System of Seed Oil Traits Using Multi-Locus Genome-Wide Association Analysis for Population Characterization and Optimal Cross Prediction in Soybean

**DOI:** 10.3389/fpls.2018.01793

**Published:** 2018-12-05

**Authors:** Yinghu Zhang, Jianbo He, Hongwei Wang, Shan Meng, Guangnan Xing, Yan Li, Shouping Yang, Jinming Zhao, Tuanjie Zhao, Junyi Gai

**Affiliations:** ^1^Soybean Research Institute, Nanjing Agricultural University, Nanjing, China; ^2^Jiangsu Coastal Institute of Agricultural Sciences, Yancheng, China; ^3^National Center for Soybean Improvement, Ministry of Agriculture, Nanjing, China; ^4^Key Laboratory of Biology and Genetic Improvement of Soybean (General), Ministry of Agriculture, Nanjing, China; ^5^State Key Laboratory for Crop Genetics and Germplasm Enhancement, Nanjing Agricultural University, Nanjing, China; ^6^Jiangsu Collaborative Innovation Center for Modern Crop Production, Nanjing Agricultural University, Nanjing, China

**Keywords:** soybean, seed oil content, oleic acid content, linolenic acid content, restricted two-stage multi-locus genome-wide association study (RTM-GWAS), SNP linkage disequilibrium block (SNPLDB), genetic differentiation, genomic selection for optimal cross

## Abstract

Soybean is one of the world's major vegetative oil sources, while oleic acid and linolenic acid content are the major quality traits of soybean oil. The restricted two-stage multi-locus genome-wide association analysis (RTM-GWAS), characterized with error and false-positive control, has provided a potential approach for a relatively thorough detection of whole-genome QTL-alleles. The Chinese soybean landrace population (CSLRP) composed of 366 accessions was tested under four environments to identify the QTL-allele constitution of seed oil, oleic acid and linolenic acid content (SOC, OAC, and LAC). Using RTM-GWAS with 29,119 SNPLDBs (SNP linkage disequilibrium blocks) as genomic markers, 50, 98, and 50 QTLs with 136, 283, and 154 alleles (2–9 per locus) were detected, with their contribution 82.52, 90.31, and 83.86% to phenotypic variance, corresponding to their heritability 91.29, 90.97, and 90.24% for SOC, OAC, and LAC, respectively. The RTM-GWAS was shown to be more powerful and efficient than previous single-locus model GWAS procedures. For each trait, the detected QTL-alleles were organized into a QTL-allele matrix as the population genetic constitution. From which the genetic differentiation among 6 eco-populations was characterized as significant allele frequency differentiation on 28, 56, and 30 loci for the three traits, respectively. The QTL-allele matrices were also used for genomic selection for optimal crosses, which predicted transgressive potential up to 24.76, 40.30, and 2.37% for the respective traits, respectively. From the detected major QTLs, 38, 27, and 25 candidate genes were annotated for the respective traits, and two common QTL covering eight genes were identified for further study.

## Introduction

Soybean [*Glycine max* (L.) Merr.] is the world's leading oilseed crop, accounting for 53.9 (29%) million metric tons of world vegetable oil consumption (http://soystats.com/international-world-vegetable-oil-consumption/,2016). The seed oil content in soybean is ~20% on average, and the oil quality is determined by the proportions of five major fatty acids, which include palmitic (C16:0), stearic (C18:0), oleic (C18:1), linoleic (C18:2), and linolenic (C18:3) acid (Wilson, 2004). The unsaturated fatty acids, such as oleic, linoleic and linolenic acids, have positive effects on human health (Bahrami, 2010), while the polyunsaturated fatty acids are not desirable for human consumption. Therefore, great efforts have been made in soybean breeding to increase the seed oil content (SOC) and oleic acid content (OAC), and to decrease the linolenic acid content (LAC) (Panthee et al., 2006).

Breeding progress depends on the potential gene resources in germplasm, such as landraces which were developed historically by farmers. The key for utilization of required genes in the germplasm population is to explore the genetic loci and alleles underlying breeding traits and to identify superior alleles. Soybean originated in China, where the crop has been cultivated for more than 5000 years (Hymowitz, 2004). During the long history, ancient Chinese farmers have developed a great number of landraces which accumulate tremendous genetic variation, and therefore, the Chinese soybean landraces are the most important gene/germplasm reservoirs for breeding programmes (Gai et al., 2012).

Soybean seed oil traits, i.e., the oil and fatty acid content, are complex traits involving a large number of genes. At present, a number of QTLs for seed oil traits have been mapped on 20 chromosomes in soybean based on linkage mapping (Supplementary Table 1). However, linkage mapping is usually applied to a segregating population derived from two parents, and therefore it is limited in terms of allelic diversity and mapping resolution (Zhu et al., 2008). The genome-wide association study (GWAS) is found to be a powerful approach to detect QTL and their multiple alleles at a relatively higher resolution, and it can also be directly applied to natural populations such as germplasm. Although a number of GWASs have been performed for soybean seed oil content (Hwang et al., 2014; Sonah et al., 2015; Wen et al., 2015; Zhou et al., 2015; Cao et al., 2017; Li et al., 2018), only few are for fatty acid composition (Li et al., 2015; Fang et al., 2017; Leamy et al., 2017).

The previous GWAS procedures concentrate on finding a handful of major loci, such as general linear model and mixed linear model (MLM) approaches (Pritchard et al., 2000b; Price et al., 2006; Yu et al., 2006) based on single-locus model, and even MLMM (Segura et al., 2013) and mrMLM (Wang et al., 2016) based on multi-locus model. But plant breeders are more likely interested in exploring the whole QTL-allele system for both forward selection and background control in breeding programs. Furthermore, the previous GWASs are generally based on SNP markers which involve only two alleles at one site, therefore the multi-allelic variation which widely exists in germplasm population cannot be detected. To overcome these limitations, He et al. (2017) proposed an innovative restricted two-stage multi-locus GWAS procedure (RTM-GWAS) for a relatively thorough detection of QTL and their multiple alleles in a germplasm population. In the RTM-GWAS procedure, the tightly linked SNPs are grouped into SNP linkage disequilibrium blocks (SNPLDBs) to form genomic markers with multiple haplotypes as alleles, and then it utilizes two-stage association analysis based on a multi-locus multi-allele model for genome-wide QTL identification along with their multiple alleles. Simulation studies demonstrated that RTM-GWAS achieved the highest QTL detection power and efficiency compared with the previous GWAS procedures, especially under large sample size and high trait heritability conditions. The RTM-GWAS procedure has been applied to identify QTL-allele system of 100-seed weight (Zhang et al., 2015) and seed isoflavone content (Meng et al., 2016) in CSLRP. More recently, Li et al. (2017) applied the RTM-GWAS procedure to a soybean nested association mapping population, and identified 139 flowering date QTLs with 496 alleles, which cover almost all QTLs detected by four other mapping procedures.

Optimal cross design and precise progeny selection are two major steps in conventional plant breeding with the former determining the potential of the latter. Peleman and van der Voort (2003) presented “Breeding by Design” concept based on QTL mapping, aiming to choose parents and design crosses for potential recombination. Meuwissen et al. (2001) proposed genomic selection (GS) as a marker-assisted selection procedure based on genome-wide SNP/markers. GS composes two links, establishing an index between required targets and SNPs/markers from a training population and then using the index in progeny selection based on its genome-wide SNP/marker information. Jonas and de Koning (2013) indicated that GS approaches from dairy cattle breeding cannot be readily applied to complex plant breeding. Therefore, following the “Breeding by Design” concept, GS based on whole-genome QTL-allele system detected from RTM-GWAS seems to be a potential approach for both optimal cross design and precise progeny selection (He et al., 2017).

In the present study, the CSLRP was used to explore QTL-allele constitutions of three major seed oil traits, i.e., SOC, OAC, and LAC, in the most important soybean gene/germplasm reservoir. Accordingly, the QTL-allele matrices were established as a compact form of the population genetic structure of seed oil traits. The matrices were used to characterize the genetic differentiation among ecoregion subpopulations and to select optimal crosses for seed oil improvement in soybean breeding. Accordingly, the candidate gene system was annotated from the detected QTLs for further study on the oil trait genes.

## Materials and Methods

### Plant Materials and Field Experiments

A sample composed of 366 soybean landraces as representative of CSLRP was used for the present study. The sampled accessions have their origination distributed in the six soybean cultivation ecoregions in China (Gai and Wang, 2001). They are I: Northern Single Cropping, Spring Planting Ecoregion; II: Huang-Huai-Hai Double Cropping, Spring, and Summer Planting Ecoregion; III: Middle and Lower Changjiang Valley Double Cropping, Spring, and Summer Planting Ecoregion; IV: South Central Multiple Cropping, Spring, Summer, and Autumn Planting Ecoregion; V: Southwest Plateau Double Cropping, Spring, and Summer Planting Ecoregion; VI: South China Tropical Multiple, All Season Planting Ecoregion (Table 1). This population has been used in the establishment of QTL-allele matrix for the 100-seed weight (Zhang et al., 2015) and seed isoflavone content (Meng et al., 2016).

**Table 1:** Frequency distribution and descriptive statistics of seed oil content, oleic acid, and linolenic acid content in CSLRP.

**Trait Env. /Eco**.		**Mid-point**										***N***	**Mean**	**Min**.	**Max**.	**GCV (%)**	***h*^2^ (%)**
**Oil content**	**<17.0**	**17.5**	**18.5**	**19.5**	**20.5**	**21.5**	**22.5**	**23.5**	**>24.0**								
Env.	08JP	4	19	49	68	103	67	25	9	5		349	20.29	15.76	24.56	6.56	89.80
	09JP	5	15	62	85	91	64	30	8	2		362	20.19	15.76	26.42	6.89	91.67
	09LS	3	16	43	96	113	67	20	7	0		365	20.19	14.95	23.57	5.74	83.44
	10JP	4	17	61	90	102	60	23	4	1		362	20.04	15.90	24.28	6.14	87.14
	Mean	1	13	52	98	109	71	18	3	1		366	20.16	15.81	24.14	5.65	91.29
Eco.	I	1	2	7	10	12	11	2	1	1		47	20.21	15.81	24.14	6.78	90.47
	II	0	2	13	22	27	14	9	0	0		87	20.29	17.18	22.98	6.51	94.01
	III	0	4	3	12	26	21	4	2	0		72	20.57	17.10	23.67	5.99	90.45
	IV	0	0	17	26	23	17	1	0	0		84	20.00	18.02	22.33	5.10	90.29
	V	0	1	4	18	9	3	2	0	0		37	19.91	17.89	22.51	4.47	86.18
	VI	0	4	8	10	12	5	0	0	0		39	19.66	17.37	21.34	5.19	89.38
Oleic acid		<15.0	16.5	19.5	22.5	25.5	28.5	31.5	34.5	37.5	40.5						
Env.	08JP	11	64	112	93	48	15	6	0	0	0	349	20.93	11.67	30.83	15.24	86.73
	09JP	27	94	93	79	43	12	5	3	5	1	362	20.53	11.07	40.44	20.65	85.82
	09LS	27	112	112	66	28	10	7	3	1	0	365	19.85	10.45	37.94	19.20	87.56
	10JP	51	102	104	63	24	11	6	1	1	0	362	19.33	10.26	36.69	19.55	85.01
	Mean	15	106	102	95	28	13	4	2	1	0	366	20.19	11.94	37.09	16.68	90.97
Eco.	I	0	6	13	16	6	4	0	1	1	0	47	22.34	15.34	37.09	18.49	90.12
	II	8	31	20	22	4	1	1	0	0	0	87	19.09	12.26	31.06	17.39	92.86
	III	3	25	19	14	6	3	2	0	0	0	72	19.86	14.23	31.25	18.93	91.58
	IV	2	19	24	29	6	3	0	1	0	0	84	20.65	11.94	34.49	16.22	89.64
	V	1	15	14	5	1	1	0	0	0	0	37	19.19	14.77	27.07	12.56	87.06
	VI	1	10	12	9	5	1	1	0	0	0	39	20.60	14.24	30.87	16.55	90.57
Linolenic acid		<3.5	4	5	6	7	8	9	10	>10.5							
Env.	08JP	0	0	4	21	83	145	71	24	1		349	7.95	5.21	10.51	11.07	89.14
	09JP	4	5	15	51	105	114	52	16	0		362	7.40	1.95	10.37	15.82	88.35
	09LS	2	1	8	16	69	150	88	28	3		365	8.03	2.99	11.61	12.96	88.98
	10JP	0	2	5	18	45	125	107	49	11		362	8.33	3.75	11.25	13.92	92.10
	Mean	2	2	3	16	94	144	81	24	0		366	7.91	2.85	10.40	11.63	90.24
Eco.	I	2	1	0	4	19	15	4	2	0		47	7.31	2.86	10.27	15.46	90.30
	II	0	0	1	4	23	29	18	12	0		87	8.07	5.00	10.10	13.01	93.56
	III	0	0	1	6	17	26	18	4	0		72	7.83	5.35	10.34	12.01	89.29
	IV	0	1	1	2	20	39	19	2	0		84	7.92	4.31	10.40	11.11	88.47
	V	0	0	0	0	8	16	13	0	0		37	8.18	6.62	9.45	7.09	78.53
	VI	0	0	0	0	7	19	9	4	0		39	8.21	6.53	10.14	10.23	89.14

*Env., environment. Eco., ecoregion, including: Ecoregion I, Northern Single Cropping, Spring Planting Ecoregion; Ecoregion II, Huang-Huai-Hai Double Cropping, Spring, and Summer Planting Ecoregion; Ecoregion III, Middle and Lower Changjiang Valleys Double Cropping, Spring, and Summer Planting Ecoregion; Ecoregion IV, South Central Multiple Cropping, Spring, Summer, and Autumn Planting Ecoregion; Ecoregion V, Southwest Plateau Double Cropping, Spring, and Summer Planting Ecoregion; Ecoregion VI, South China Tropical Multiple, All Season Planting Ecoregion. GCV, genotypic coefficient of variation; h^2^, trait heritability value estimated from ANOVA*.

The materials were tested in randomized complete block design (RCBD) experiments, 0.7 m × 0.8 m hill plots with two replications at Jiangpu Experimental Station (abbreviated as JP) of Nanjing Agricultural University, Nanjing, China in 2008, 2009, and 2010, and two replications at Lishui Experimental Station (abbreviated as LS), Nanjing, China in 2009. The hill plots were thinned to six seedlings per plot (Wen et al., 2009). The planting dates were 20 June 2008JP, 19 June 2009JP, 26 June 2009LS, and 23 June 2010JP, where the codes of 2008JP, 2009JP, 2010JP, and 2009LS represent the environments composed of the year (2008–2010) and location (JP and LS), respectively.

A specimen of 20 g seeds for each replication, each accession of the CSLRP were milled with a 1095 Knifetec sample mill (FOSS Tecator, Denmark), then NIR spectroscopy analysis was performed using VECTOR22/N (BRUKER, German), and finally the SOC, OAC, LAC were converted using the calibration model developed by Wang (2011).

### Statistical Analysis

A joint analysis of variance (ANOVA) was conducted for the CSLRP using PROC GLM of SAS 9.4 (SAS Institute Inc., Cary, NC, USA), in which the genotype, environment, replication, and genotype-by-environment interaction were considered to be random effects. The heritability (*h*^2^) was estimated as ${\hat{h}}^{2}={\hat{\sigma }}_{g}^{2}/\left({\hat{\sigma }}_{g}^{2}+{\hat{\sigma }}^{2}/r\right)$ for individual environments and ${\hat{h}}^{2}={\hat{\sigma }}_{g}^{2}/\left[{\hat{\sigma }}_{g}^{2}+{\hat{\sigma }}_{ge}^{2}/s+\sigma^{2}/\left(sr\right)\right]$ for multi-environment joint analysis, where ${\hat{\sigma }}_{g}^{2}$, ${\hat{\sigma }}_{ge}^{2}$ and ${\hat{\sigma }}^{2}$ are estimated variances of genotype, genotype-by-environment interaction, and the random error, respectively, and *s* is the number of environments and *r* is the number of replications in an experiment (Hanson et al., 1956). The variance components were estimated using REML method with PROC VARCOMP of SAS 9.4. The genetic coefficient of variation was calculated as ${\hat{\sigma }}_{g}/\mu$ , where μ is the population mean.

### Genotyping

The RAD-Seq (restriction site-associated DNA sequencing) was used for SNP genotyping in the present study. All the genotyping work was done at BGI Tech, Shenzhen, China. A total of 116,769 SNPs were identified after quality control and grouped into 29,119 SNP linkage disequilibrium blocks (SNPLDBs) according to He et al. (2017), Zhang et al. (2015), and Meng et al. (2016). The SNPLDB is a segment with its SNPs linked together. The sequence of each SNPLDB/segment differentiated among the 366 landraces and formed haplotypes in the same region which were considered to be alleles on a same locus/SNPLDB.

### Association Mapping

Association mapping was conducted with the innovative restricted two-stage multi-locus GWAS (RTM-GWAS) procedure (He et al., 2017). At the first stage, single-locus association test based on the simple linear model was used to eliminate redundant markers, and at the second stage, the stepwise regression was applied to build the final multi-locus model based on candidate markers pre-selected from the first stage. The top 10 eigenvectors (accounting for 86% of the total variation) of the genetic similarity coefficient matrix built on SNPLDBs were incorporated as covariates to correct for population structure.

Since the environment factor in the present study involved 3 years and two locations in a same city which did not relate to certain fixed factors, therefore, the whole set of the data rather than individual environment data were used for association mapping. The mean data set across all environments were used for association analysis with a normal significance level of 0.02 as the built-in control for experiment-wise error rate of multi-locus model. As more stringent significance levels, such as 0.0002, were also suggested in other multi-locus methods such as mrMLM. Therefore, to identify candidate genes corresponding to major QTLs, a significance level of 0.0002 was also used in RTM-GWAS.

To compare the results with the previous GWAS methods, the MLM GWAS were also performed. The population structure matrix (Q) estimated from STRUCTURE 2.2 (Pritchard et al., 2000a) and the familial relatedness matrix (K) were used jointly, and the association analysis was performed using TASSEL software (Bradbury et al., 2007).

### Genetic Differentiation Analysis

The analysis of molecular variance (AMOVA) for molecular variance among ecoregions was carried out based on the whole genome SNPLDBs and the SNPLDBs associated with the seed oil traits, respectively. The Arlequin 3.5.2.2 software were used for the computations (Excoffier and Lischer, 2010). To examine the difference among the ecoregion QTL-allele matrices, the chi-squared test was used to test the independence of the allele frequency distribution among ecoregions for each locus using the PROC FREQ in SAS.

### Optimal Cross Prediction

All possible single crosses among the 366 accessions (66,795) were generated *in silico* under linkage model and independent assortment model for recombination potential of the seed oil traits (He et al., 2017). For each cross, the predicted genotypic value of seed oil traits was calculated based on 2,000 continuously inbred progenies derived from F_1_ individuals and the QTL-allele matrix. The 99th percentile of a cross was used as its predicted cross value for SOC and OAC, and the 1st percentile of a cross was used as its predicted cross value for LAC.

### Candidate Gene Annotation

From the detected QTL system in CSLRP, the candidate genes were annotated using the following steps: firstly, the genes located <100 kb away from the associated SNPLDBs were identified based on SoyBase (http://soybase.org); secondly, those genes containing SNPs in the population were identified; and finally, those genes containing SNPs significantly associated with the detected SNPLDB through chi-square test were considered as candidate genes. The annotations and expression data of candidate genes were retrieved from SoyBase (http://soybase.org), and the genes were grouped into three categories, i.e., biological process, cellular component, and molecular function. The pathway analysis of candidate genes was performed based on SoyCyc Soybean Metabolic Pathway Database (https://soycyc.soybase.org).

## Results

### Phenotypic and Genotypic Variation of Seed Oil Traits in the CSLRP

There is a wide variation of the seed oil traits in the CSRLP, ranging from 14.95 to 26.42%, 10.26 to 40.44%, and 1.95 to 11.61% for SOC, OAC, and LAC, respectively (Table 1). The heritability of SOC, OAC, and LAC were estimated as 91.29, 90.97, and 90.24% with the genetic coefficient of variation ranging from 5.65 to 16.68. The results from ANOVA showed significant genotype-by-environment interactions, while the genotypic variation among the landraces was 10.24 (LAC) to 11.78 (SOC) times of that of genotype-by-environment interaction in CSLRP (Supplementary Table 2).

The whole sample was separated into six sub-samples according to ecoregions in China (Table 1). The phenotype differences of the seed oil traits among ecoregion means were not large, but large phenotype variation within ecoregion existed, indicating abundant variation in each ecoregion. Among ecoregions, the varieties from ecoregion I (Northeast China) have relatively more SOC and OAC, but less LAC. That might be due to the enhancement of soybean improvement on seed oil traits in this region during the recent decades. Since large variation existed in each ecoregion and the soybean landraces were developed independently by local farmers, exploring QTL-allele constitutions of each ecoregion may provide information on genetic improvement potential of the three oil traits.

### The QTL Systems of Seed Oil Traits in the CSRLP

**Figure 1:**
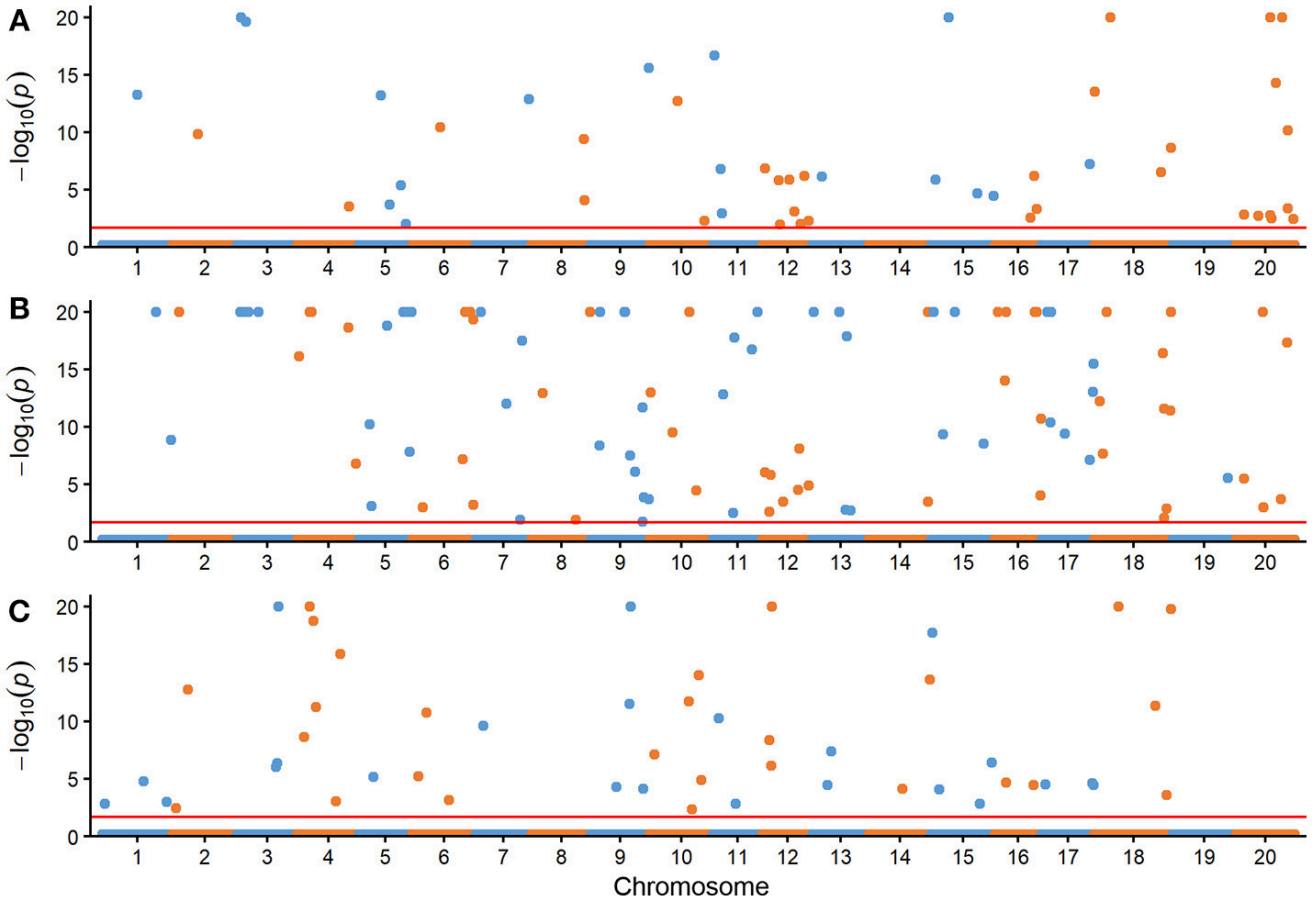
Manhattan plots of three seed oil traits with the RTM-GWAS method. **(A)** Seed oil content (%), **(B)** oleic acid content (%) in seed oil, **(C)** linolenic acid content (%) in seed oil. The –log_10_(*P*) was set to 20 if it was >20. The horizontal dotted lines represent the genome-wide threshold.

The comparison for the number of significantly associated markers obtained from RTM-GWAS and MLM was summarized in Table 2. If correction for multiple testing is not considered (using a normal significance level of 0.05), the MLM method showed tremendous overflowing (*R*^2^ > 100%) of the total phenotypic contribution. But in contrast, a large amount of missing heritability in comparison with the trait heritability (especially for SOC) was observed for the MLM method under the Bonferroni-adjusted significance level (Supplementary Figure 1). However, with the RTM-GWAS method which was based on multi-locus association analysis, a total of 50, 98, and 50 SNPLDBs were detected to be significantly (under a significance level of 0.02) associated with SOC, OAC, and LAC, respectively (Figure 1). The total contribution to phenotype variance of the associated loci were 82.53, 90.29, and 83.84%, which were close to but did not exceed the trait heritability values, 91.29, 90.97, and 90.24%, respectively (Table 2). A more stringent significance level of 0.0002 was also used in RTM-GWAS, and 13, 19, and 12 in a total of 42 SNPLDBs (two loci overlap) were identified for SOC, OAC, and LAC, respectively. As expected, the SNPLDBs detected using stringent significance level were included in the SNPLDBs under normal significance level with the same *p*-value order, except that two SNPLDBs for SOC were newly detected using 0.0002 (Tables 3, 4, Supplementary Table 3). The loci excluded due to stringent significance level were mainly small-contribution loci (*R*^2^ < 1%), and the total contribution to phenotype variance decreased slightly to 55.38, 67.47, and 56.63%, respectively. Therefore, major loci can be identified directly from RTM-GWAS results without recalculation using a stringent significance level.

**Table 2:** Comparisons of the GWAS results of the seed oil traits in CSLRP among the two association analysis procedures.

**Trait**	***h*^2^ (%)**	**RTM-GWAS**				**MLM**			
		**Normal** **(0.02)**		**Stringent** **(0.0002)**		**Normal** **(0.05)**		**Bonferroni** **(0.05/m** = **1.7e-6)**	
		**No**.	***R*^2^ (%)**	**No**.	***R*^2^ (%)**	**No**.	***R*^2^ (%)**	**No**.	***R*^2^ (%)**
Oil content	91.29	50	82.53	13	55.38	2,405	4482.78	3	19.69
Oleic acid	90.97	98	90.29	19	67.47	2,873	5769.33	18	138.76
Linolenic acid	90.24	50	83.84	12	56.63	2,574	5132.79	22	206.52

*No., the number of SNPLDBs detected to be significantly associated with a trait. R^2^, total phenotype contribution calculated as the sum of R^2^-value of each significantly associated locus. Normal (0.02) and Normal (0.05) represent two normal significance levels of 0.02 and 0.05, respectively. Stringent (0.0002) represents the stringent significance level of 0.0002 as suggested in mrMLM. Bonferroni (0.05/m = 1.7e-6) represents the Bonferroni-adjusted significance level of 0.05, i.e., 0.05 divided by the number of markers (m = 29,119)*.

**Table 3:** The detected SNPLDBs associated with seed oil content in CSLRP.

**QTL**	**SNPLDB**	**Allele number**	**–log_10_*P***	***R*^2^**	**SoyBase QTL**
*Oil-a-01-1*	Gm01_28027741	2	13.26	2.06	*C-Oil-01-1*
*Oil-a-02-1*	Gm02_20124502	2	9.81	1.44	
*Oil-a-03-1*	Gm03_BLOCK36_2969353_2978158	3	**24.55**	4.58	*C-Oil-03-1*
*Oil-a-03-2*	Gm03_6682794	2	**19.63**	3.29	*C-Oil-03-1*
*Oil-a-04-1*	Gm04_BLOCK329_41069142_41069353	2	3.52	0.44	
*Oil-a-05-1*	Gm05_17497538	2	13.20	2.04	
*Oil-a-05-2*	Gm05_24316463	2	3.71	0.47	*C-Oil-05-2*
*Oil-a-05-3*	Gm05_33053483	2	5.36	0.72	*C-Oil-05-3*
*Oil-a-05-4*	Gm05_BLOCK179_37315189_37334388	2	1.99	0.22	*C-Oil-05-3*
*Oil-a-06-1*	Gm06_BLOCK186_22452807_22652100	5	10.43	1.97	*C-Oil-06-3*
*Oil-a-07-1*	Gm07_BLOCK336_42734434_42793375	3	12.85	2.16	*C-Oil-07-2*
*Oil-a-08-1*	Gm08_41719184	2	9.41	1.38	*C-Oil-08-3*
*Oil-a-08-2*	Gm08_41878014	2	4.06	0.52	*C-Oil-08-3*
*Oil-a-09-1*	Gm09_46185913	2	**15.57**	2.48	*C-Oil-09-2*
*Oil-a-10-1*	Gm10_22537585	2	**12.71**	1.95	
*Oil-a-10-2*	Gm10_BLOCK348_44121054_44124979	2	2.30	0.26	
*Oil-a-11-1*	Gm11_BLOCK8_937574_956972	4	**16.65**	3.06	
*Oil-a-11-2*	Gm11_BLOCK43_6132671_6135584	3	6.76	1.08	*C-Oil-11-1*
*Oil-a-11-3*	Gm11_7192431	2	2.95	0.35	*C-Oil-11-1*
*Oil-a-12-1*	Gm12_BLOCK24_2388452_2413507	2	6.82	0.95	
*Oil-a-12-2*	Gm12_13269224	2	5.80	0.79	*C-Oil-12-1*
*Oil-a-12-3*	Gm12_BLOCK105_14154928_14154934	3	1.97	0.30	*C-Oil-12-1*
*Oil-a-12-4*	Gm12_21507884	2	5.88	0.80	
*Oil-a-12-5*	Gm12_25545925	2	3.12	0.38	
*Oil-a-12-6*	Gm12_30236411	2	2.03	0.22	
*Oil-a-12-7*	Gm12_BLOCK165_33232631_33233256	2	6.20	0.85	*C-Oil-12-2*
*Oil-a-12-8*	Gm12_36993179	2	2.31	0.26	*C-Oil-12-2*
*Oil-a-13-1*	Gm13_7202546	2	6.14	0.84	*C-Oil-13-1*
*Oil-a-15-1*	Gm15_BLOCK26_3744298_3852076	3	5.86	0.93	*C-Oil-15-1*
*Oil-a-15-2*	Gm15_BLOCK102_13882165_13885992	3	**23.10**	4.25	*C-Oil-15-1*
*Oil-a-15-3*	Gm15_BLOCK270_37041976_37241042	9	4.68	1.23	*C-Oil-15-2*
*Oil-a-15-4*	Gm15_BLOCK395_49963243_49997145	3	4.46	0.70	*C-Oil-15-2*
*Oil-a-16-1*	Gm16_28573322	2	2.55	0.30	*C-Oil-16-3*
*Oil-a-16-2*	Gm16_BLOCK285_31506333_31515376	4	6.21	1.09	*C-Oil-16-3*
*Oil-a-16-3*	Gm16_BLOCK302_33491699_33540765	4	3.33	0.60	
*Oil-a-17-1*	Gm17_BLOCK296_38209773_38210321	2	7.21	1.01	*C-Oil-17-3*
*Oil-a-18-1*	Gm18_470696	2	**13.52**	2.10	
*Oil-a-18-2*	Gm18_BLOCK123_12806957_13006775	7	**31.62**	7.17	
*Oil-a-18-3*	Gm18_53216372	2	6.54	0.90	*C-Oil-18-2*
*Oil-a-18-4*	Gm18_61147465	2	8.65	1.25	*C-Oil-18-3*
*Oil-a-20-1*	Gm20_6366862	2	2.85	0.34	*C-Oil-20-1*
*Oil-a-20-2*	Gm20_17824845	2	2.73	0.32	*C-Oil-20-1*
*Oil-a-20-3*	Gm20_BLOCK144_27228799_27268887	6	**43.62**	10.60	*C-Oil-20-1*
*Oil-a-20-4*	Gm20_27461408	2	2.80	0.33	*C-Oil-20-1*
*Oil-a-20-5*	Gm20_28265310	2	2.50	0.29	*C-Oil-20-1*
*Oil-a-20-6*	Gm20_BLOCK180_31674614_31694438	2	**14.29**	2.25	*C-Oil-20-1*
*Oil-a-20-7*	Gm20_BLOCK240_36660853_36753656	6	**38.04**	8.82	*C-Oil-20-1*
*Oil-a-20-8*	Gm20_41299844	2	10.14	1.50	*C-Oil-20-2*
*Oil-a-20-9*	Gm20_41428832	2	3.36	0.41	*C-Oil-20-2*
*Oil-a-20-10*	Gm20_46016274	2	2.46	0.28	*C-Oil-20-3*
LC QTL		77		23 (68.77)	17 (14)
SC QTL		59		27 (13.76)	20 (14)
Total		136		50 (82.53)	37 (21)

*LC QTL and SC QTL: large-contribution (R^2^ ≥ 1%) and small-contribution (R^2^ < 1%) QTL, respectively; SNPLDB: abbreviation of SNP linkage disequilibrium block, that is designated as Gm03_BLOCK36_2969353_2978158 if it contains multiple SNPs where Gm03 denotes Chromosome 3, BLOCK36 denotes 36th SNPLDB on the Chromosome, and 2969353_2978158 is its physical position in bp; that is designated as Gm01_28027741 if it contains only one SNP where Gm01 denotes Chromosome 1, and 28027741 is its physical position in bp. In column R^2^, 50 (82.53) means that a total of 50 QTL were detected which explained 82.53% of the phenotype variation. In column SoyBase QTL, 37 (21) denotes that 37 QTL detected through association mapping were located in or around the 21 different QTL reported in SoyBase (http://soybase.org). The –log_10_P-value in bold style means that the locus is also identified with a stringent significance level of 0.0002, and the total contribution to phenotype variation is 55.38%*.

**Table 4:** The detected SNPLDBs associated with oleic acid and linolenic acid content in CSLRP.

**QTL**	**SNPLDB**	**Allele number**	**Oleic acid**		**Linolenic acid**		**SoyBase QTL**
			**–log_10_*P***	***R*^2^**	**–log_10_*P***	***R*^2^**	
*Oleic-a-01-1*	Gm01_BLOCK282_42690478_42711941	4	**63.43**	3.14			
*Oleic-a-02-1*	Gm02_5111631	2	**87.43**	6.19			
*Linolenic-a-02-2*	Gm02_12208888	2			**12.75**	1.54	*C-Linolenic-02-1*
*Linolenic-a-03-3*	Gm03_BLOCK254_32437120_32636442	5			**47.45**	9.40	
*Oleic-a-04-2 /Linolenic-a-04-2*	Gm04_BLOCK96_9670209_9789832	7	**108.81**	13.20	**66.74**	16.83	*C-Linolenic-04-1*
*Oleic-a-04-3*	Gm04_BLOCK112_11374739_11441096	9	**82.13**	6.35			
*Linolenic-a-04-3*	Gm04_12902712	2			**18.71**	2.45	
*Linolenic-a-04-4*	Gm04_BLOCK138_14758735_14783243	7			11.25	1.85	
*Linolenic-a-04-6*	Gm04_34223987	2			**15.87**	2.00	
*Oleic-a-05-4*	Gm05_BLOCK156_35227925_35227954	2	**55.11**	2.21			*C-Oleic-05-1*
*Oleic-a-05-5*	Gm05_37451040	2	**40.40**	1.27			*C-Oleic-05-1*
*Oleic-a-05-7*	Gm05_41857301	2	**45.37**	1.54			*C-Oleic-05-2*
*Linolenic-a-06-2*	Gm06_11505878	2			10.78	1.27	
*Oleic-a-06-3*	Gm06_42628531	2	**37.46**	1.12			
*Oleic-a-06-4*	Gm06_46292942	2	**55.84**	2.26			
*Oleic-a-07-1*	Gm07_4223914	2	**49.99**	1.84			
*Linolenic-a-07-1*	Gm07_BLOCK47_6321758_6323896	3			9.62	1.23	
*Oleic-a-08-3*	Gm08_46400798	2	**42.26**	1.37			
*Oleic-a-09-2*	Gm09_BLOCK84_7712817_7796042	5	36.56	1.22			*C-Oleic-09-1*
*Oleic-a-09-3*	Gm09_BLOCK222_26785007_26956034	3	**44.62**	1.57			
*Linolenic-a-09-2*	Gm09_30906609	2			11.50	1.37	
*Linolenic-a-09-3*	Gm09_BLOCK255_31868217_31984435	6			**35.28**	6.40	*C-Linolenic-09-2*
*Linolenic-a-10-2*	Gm10_BLOCK232_31403627_31521273	5			11.75	1.75	
*Linolenic-a-10-4*	Gm10_39620012	2			**14.00**	1.72	
*Linolenic-a-11-1*	Gm11_4666410	2			10.24	1.19	
*Linolenic-a-12-3*	Gm12_7530543	2			**23.87**	3.32	
*Oleic-a-13-2*	Gm13_21132878	2	**73.55**	4.05			*C-Oleic-13-1*
*Oleic-a-14-1*	Gm14_BLOCK392_47269359_47316131	5	**54.43**	2.39			
*Linolenic-a-14-2*	Gm14_49054789	2			**13.63**	1.67	*C-Linolenic-14-1*
*Linolenic-a-15-1*	Gm15_1215528	2			**17.68**	2.28	*C-Linolenic-15-1*
*Oleic-a-15-1*	Gm15_2169757	2	**42.55**	1.38			*C-Oleic-15-1*
*Oleic-a-15-3*	Gm15_BLOCK141_18940958_19140709	5	**84.29**	6.18			*C-Oleic-15-1*
*Oleic-a-16-1*	Gm16_2601283	2	**44.32**	1.48			
*Oleic-a-16-3*	Gm16_9334192	2	36.50	1.07			
*Oleic-a-17-1*	Gm17_4601421	2	**82.39**	5.32			
*Linolenic-a-18-1*	Gm18_BLOCK171_19084763_19284540	7			**33.77**	6.22	
*Linolenic-a-18-2*	Gm18_48712671	2			11.38	1.35	
*Oleic-a-18-9 /Linolenic-a-18-4*	Gm18_BLOCK530_61189911_61190505	3	**76.52**	4.61	**19.78**	2.79	
LC QTL		67/65	21 (69.76)		19 (66.63)		7/5
SC QTL		216/89	77 (20.53)		31 (17.21)		11/4
Total		283/154	98 (90.29)		50 (83.84)		18/9

*LC QTL and SC QTL: large-contribution (R^2^ ≥ 1%) and small-contribution (R^2^ < 1%) QTL, respectively; QTL in the left of “/” was the oleic acid content QTL while the right was the linolenic acid content QTL. The –log_10_P-value in bold style means that the locus is also identified with a stringent significance level of 0.0002. The detailed SC QTL was listed in Supplementary Table 4*.

Among the detected QTLs of the three seed oil traits, 23, 21, and 19 large-contribution QTLs (*R*^2^ ≥ 1%) explained a total of 68.77, 69.76, and 66.63% of phenotypic variation, while 27, 77, and 31 small-contribution QTLs (*R*^2^ < 1%) explained a total of 13.76, 20.53, and 17.21% of phenotypic variation, respectively (Tables 3, 4, Supplementary Table 4). In the SOC QTL system, 50 QTLs were detected with each QTL contribution to phenotypic variance varied continuously and greatly from 0.26 to 10.60%, and a group of minor QTLs were detected collectively but not individually with a total contribution of 8.76%. The SOC QTL system composed of both individually and collectively detected QTLs, and similar phenomenon was also observed for OAC and LAC. This indicated that the three seed oil traits are genetically complex traits, and there are great potentials in the improvement of the three oil traits through genetic recombination. Therefore, how to converge all or most of the elite alleles through breeding procedures is to be explored.

The SOC QTLs distributed on 18 chromosomes except Gm14 and Gm19 (Supplementary Table 5). There were 1-10 QTLs located on each chromosome. Gm20 harbored 10 QTL, with a total phenotypic contribution of 25.15%, indicating its genetic importance for oil content. The OAC QTLs distributed on all 20 chromosomes, also with 1-10 QTLs located on each chromosome. Gm09 harbored 10 QTLs, explaining a total of 4.45% of phenotypic variation, while Gm04 had 5 QTLs, explaining a total of 20.46% of phenotypic variation, suggesting Gm04 being of genetic importance in determining oleic content. The LAC QTLs distributed on 17 chromosomes except Gm08, Gm19, and Gm20. There were 1-6 QTLs located on each chromosome, and Gm04 had 6 QTLs, explaining a total of 24.66% of phenotypic variation, suggesting Gm04 being of genetic importance in determining linolenic content.

### The QTL-Allele Matrices of Seed Oil Traits of the CSLRP

**Figure 2:**
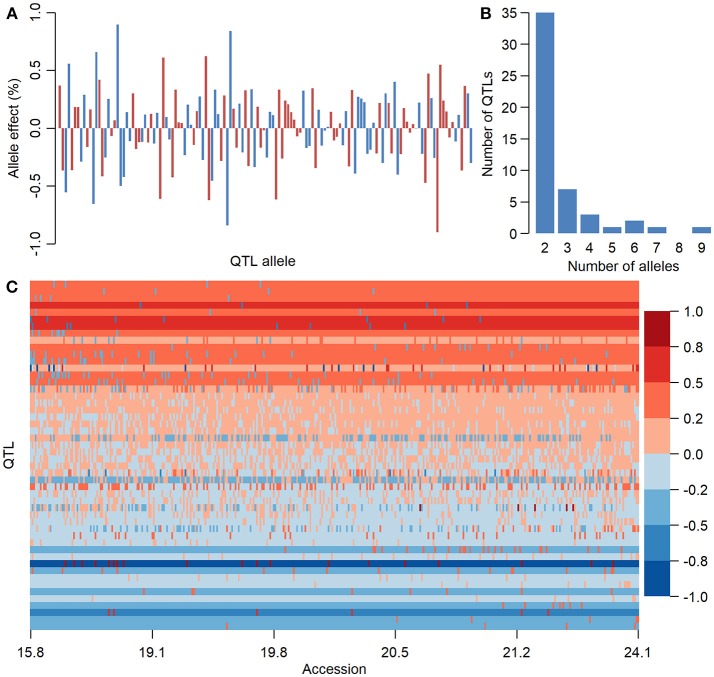
The information of seed oil content QTL-allele matrix of CSLRP. **(A)** Effect distribution of 136 alleles on the 50 loci for seed oil content in CSLRP. A bar represents an allele. **(B)** Distribution of number of alleles on the 50 loci for seed oil content (%). **(C)** Graphical representation of seed oil content QTL-allele matrix of CSLRP. The horizontal axis represents accessions arranged in a rising order of their oil content (%), while the vertical axis represents QTL arranged in a rising order of their positive allele frequency. Each row represents the allele distribution among accessions for a QTL, while each column indicates the allele constitution of an accession over all QTLs. Allele effects are expressed in color cells with warm colors indicating positive effects and cool colors indicating negative effects, and the color depth indicates effect size.

There were 136, 283, and 154 alleles on 50, 98, and 50 QTLs of SOC, OAC, and LAC, respectively, with their allele effects estimated from RTM-GWAS (Supplementary Table 6). The QTL-allele effects in the 366 Chinese soybean landraces can be organized into 136 × 366, 283 × 366, and 154 × 366 (allele × accession) matrices which were designated as QTL-allele matrix of SOC, OAC, and LAC of CSLRP, respectively. This QTL-allele matrix contains all the genetic information of the population and in fact is a matrix of the genetic constitution of the population. Figure 2 showed a compact form of the 50 × 366 (locus × accession) SOC QTL-allele matrix of CSLRP with allele effects presented in color gradient. Each row represents the allele distribution among accessions for a QTL, while each column indicates the allele constitution of an accession over all QTLs. It can be found that no landrace contains alleles that are all with negative or positive effect on the 50 loci, and lines of high oil content have more alleles with positive effect. The population contains mainly alleles with positive effect for QTLs at the top, and the alleles with negative effect are in only several lines. In contrast, the population contains mainly alleles with negative effect for QTLs at the bottom. The OAC and LAC QTL-allele matrices showed similar patterns (Supplementary Figures 2, 3).

The QTL-allele constitution of 10 lowest and highest SOC landraces were listed in Table 5. It can be found that the two landrace groups shared same alleles on nine loci (five alleles with positive effect and four alleles with negative effect), and differentiation existed on 41 loci. The total number of alleles with positive effect in the high-SOC group was 295 (ranging from 27 to 33 with an average of 29.5 per landrace), while was 227 in the low-SOC group (ranging from 19 to 25 with an average of 22.7 per landrace). The landrace N24274 with the highest SOC of 24.1% composed of 31 and 19 alleles with positive and negative effect, respectively, while the landrace N24600 with the lowest SOC of 15.8% composed of 20 and 30 alleles with positive and negative effect, respectively. Similar phenomenon was observed for OAC and LAC (Supplementary Tables 7, 8). Therefore, for the three seed oil traits, a great potential in recombination breeding may be achieved if redundant linkage can be broken. Furthermore, some alleles with positive effect only existed in accessions with the highest SOC, while some alleles with negative effect were only in accessions with the lowest SOC. For example, the 6-th allele of locus *Oil-a-20-7* with an effect of −0.9 only existed in accessions with the lowest SOC, while the 3-th allele with an effect of 0.5 only existed in accessions with the highest SOC (Table 5). Similar results were observed for OAC and LAC. This indicated that in addition to the accumulation of alleles with positive effect, emergence of elite alleles may be another way in the improvement of the seed oil traits.

**Table 5:** QTL-allele constitution of twenty accessions with the lowest and highest seed oil content in CSLRP.

**QTL**	***R*^2^**	**Lowest seed oil content**										**Highest seed oil content**									
		**N24600**	**N24609**	**N20941**	**N05410.2**	**N02806**	**N23632**	**N24619**	**N24608**	**N24610**	**N24281**	**N24293**	**N23591**	**N23598**	**N23592**	**N24452**	**N01842.1**	**N23533**	**N23602**	**N05283.2**	**N24274**
*Oil-a-01-1*	2.1	0.4	0.4	0.4	−0.4	0.4	0.4	0.4	0.4	0.4	0.4	0.4	0.4	0.4	0.4	0.4	0.4	0.4	0.4	0.4	0.4
*Oil-a-02-1*	1.4	0.6	−0.6	0.6	0.6	0.6	0.6	0.6	0.6	0.6	0.6	0.6	0.6	0.6	0.6	0.6	0.6	0.6	0.6	0.6	0.6
*Oil-a-03-1*	4.6	0.2	0.2	0.2	−0.4	0.2	0.2	0.2	0.2	0.2	0.2	0.2	0.2	0.2	0.2	0.2	0.2	0.2	0.2	0.2	0.2
*Oil-a-03-2*	3.3	−0.3	−0.3	−0.3	−0.3	−0.3	−0.3	−0.3	−0.3	−0.3	−0.3	−0.3	−0.3	−0.3	−0.3	−0.3	−0.3	−0.3	−0.3	−0.3	−0.3
*Oil-a-04-1*	0.4	0.2	0.2	−0.2	−0.2	−0.2	−0.2	0.2	−0.2	−0.2	−0.2	−0.2	−0.2	−0.2	−0.2	−0.2	−0.2	−0.2	−0.2	−0.2	−0.2
*Oil-a-05-1*	2.0	−0.7	0.7	0.7	0.7	0.7	0.7	0.7	0.7	0.7	0.7	0.7	0.7	0.7	0.7	0.7	0.7	0.7	0.7	0.7	0.7
*Oil-a-05-2*	0.5	−0.4	−0.4	−0.4	−0.4	−0.4	−0.4	−0.4	−0.4	−0.4	−0.4	−0.4	−0.4	−0.4	−0.4	−0.4	−0.4	−0.4	−0.4	−0.4	−0.4
*Oil-a-05-3*	0.7	−0.3	0.3	−0.3	−0.3	−0.3	−0.3	−0.3	−0.3	−0.3	−0.3	−0.3	−0.3	−0.3	−0.3	−0.3	−0.3	−0.3	−0.3	−0.3	−0.3
*Oil-a-05-4*	0.2	−0.1	−0.1	−0.1	−0.1	−0.1	0.1	0.1	−0.1	−0.1	−0.1	0.1	0.1	0.1	−0.1	−0.1	−0.1	−0.1	0.1	0.1	0.1
*Oil-a-06-1*	2.0	−0.1	−0.4	0.1	−0.1	−0.4	−0.5	0.1	−0.4	0.1	0.1	0.1	−0.1	−0.1	−0.1	−0.1	−0.1	−0.1	−0.1	−0.1	−0.1
*Oil-a-07-1*	2.2	0.3	0.3	0.3	−0.1	−0.1	−0.1	0.3	−0.1	0.3	0.3	0.3	0.3	0.3	0.3	0.3	−0.1	−0.2	0.3	0.3	0.3
*Oil-a-08-1*	1.4	−0.1	−0.1	−0.1	0.1	−0.1	−0.1	−0.1	−0.1	0.1	0.1	0.1	−0.1	−0.1	−0.1	−0.1	0.1	0.1	0.1	0.1	0.1
*Oil-a-08-2*	0.5	0.1	0.1	0.1	−0.1	0.1	0.1	0.1	0.1	0.1	−0.1	0.1	0.1	0.1	0.1	0.1	0.1	0.1	−0.1	−0.1	−0.1
*Oil-a-09-1*	2.5	−0.1	0.1	−0.1	0.1	−0.1	0.1	−0.1	0.1	−0.1	−0.1	0.1	−0.1	−0.1	−0.1	−0.1	0.1	−0.1	0.1	0.1	0.1
*Oil-a-10-1*	2.0	0.6	0.6	0.6	0.6	0.6	0.6	0.6	0.6	0.6	0.6	0.6	0.6	0.6	0.6	0.6	0.6	0.6	0.6	0.6	0.6
*Oil-a-10-2*	0.3	0.1	0.1	0.1	−0.1	−0.1	0.1	−0.1	0.1	−0.1	−0.1	0.1	0.1	0.1	0.1	0.1	0.1	0.1	−0.1	0.1	0.1
*Oil-a-11-1*	3.1	−0.4	0.0	−0.4	0.1	0.1	0.3	0.1	−0.4	−0.4	0.1	0.1	−0.4	−0.4	0.3	0.3	0.1	0.1	0.1	0.3	0.3
*Oil-a-11-2*	1.1	0.0	−0.2	0.0	−0.2	0.0	−0.2	−0.2	−0.2	−0.2	−0.2	−0.2	−0.2	−0.2	−0.2	−0.2	−0.2	−0.2	−0.2	−0.2	−0.2
*Oil-a-11-3*	0.4	−0.1	0.1	−0.1	−0.1	−0.1	−0.1	−0.1	−0.1	−0.1	−0.1	−0.1	0.1	0.1	−0.1	0.1	0.1	0.1	−0.1	−0.1	−0.1
*Oil-a-12-1*	0.9	−0.3	−0.3	−0.3	0.3	0.3	0.3	0.3	0.3	−0.3	−0.3	0.3	0.3	0.3	0.3	0.3	0.3	0.3	0.3	0.3	0.3
*Oil-a-12-2*	0.8	−0.6	−0.6	−0.6	−0.6	−0.6	−0.6	−0.6	−0.6	−0.6	−0.6	−0.6	−0.6	−0.6	−0.6	−0.6	−0.6	−0.6	−0.6	−0.6	−0.6
*Oil-a-12-3*	0.3	0.1	0.1	0.1	0.1	0.1	0.1	0.1	0.1	0.1	0.1	0.1	0.1	0.3	0.1	0.1	0.1	0.1	0.1	0.1	0.1
*Oil-a-12-4*	0.8	−0.3	−0.3	−0.3	−0.3	−0.3	−0.3	−0.3	−0.3	−0.3	−0.3	−0.3	−0.3	−0.3	−0.3	−0.3	−0.3	−0.3	0.3	−0.3	−0.3
*Oil-a-12-5*	0.4	−0.8	−0.8	−0.8	−0.8	−0.8	−0.8	−0.8	−0.8	−0.8	−0.8	−0.8	−0.8	−0.8	−0.8	−0.8	−0.8	−0.8	−0.8	−0.8	−0.8
*Oil-a-12-6*	0.2	−0.2	−0.2	−0.2	−0.2	−0.2	−0.2	−0.2	−0.2	−0.2	−0.2	−0.2	−0.2	−0.2	−0.2	−0.2	−0.2	−0.2	−0.2	−0.2	−0.2
*Oil-a-12-7*	0.8	0.2	0.2	0.2	−0.2	0.2	0.2	0.2	0.2	0.2	0.2	0.2	0.2	0.2	0.2	0.2	0.2	0.2	0.2	0.2	0.2
*Oil-a-12-8*	0.3	0.3	0.3	0.3	0.3	0.3	0.3	0.3	0.3	0.3	0.3	0.3	0.3	0.3	0.3	0.3	0.3	0.3	0.3	0.3	0.3
*Oil-a-13-1*	0.8	−0.3	0.3	−0.3	0.3	0.3	0.3	0.3	0.3	0.3	−0.3	0.3	0.3	0.3	0.3	0.3	0.3	0.3	0.3	0.3	0.3
*Oil-a-15-1*	0.9	0.0	0.0	0.0	−0.2	0.2	0.0	0.0	−0.2	0.0	0.0	0.2	0.2	0.2	0.2	0.2	0.2	0.2	0.2	−0.2	−0.2
*Oil-a-15-2*	4.3	−0.3	−0.3	−0.3	0.1	0.1	−0.3	−0.3	−0.3	−0.3	0.1	0.1	0.1	0.1	0.1	−0.3	−0.3	0.1	−0.3	0.1	0.1
*Oil-a-15-3*	1.2	0.0	0.1	0.0	0.1	0.1	0.1	0.1	0.1	0.0	0.0	0.0	0.1	−0.1	−0.1	0.1	0.3	0.0	0.3	0.0	0.0
*Oil-a-15-4*	0.7	−0.2	−0.2	−0.2	−0.2	−0.2	−0.2	−0.2	−0.2	−0.2	−0.2	0.3	−0.2	−0.2	−0.2	−0.2	−0.2	−0.2	−0.2	−0.2	−0.2
*Oil-a-16-1*	0.3	0.3	0.3	0.3	−0.3	0.3	0.3	−0.3	0.3	0.3	0.3	0.3	0.3	0.3	0.3	0.3	0.3	0.3	0.3	0.3	0.3
*Oil-a-16-2*	1.1	−0.1	0.0	0.2	−0.1	−0.1	−0.1	−0.1	−0.1	−0.1	−0.1	0.2	−0.1	0.0	−0.1	−0.1	−0.1	0.0	−0.1	0.0	0.0
*Oil-a-16-3*	0.6	0.1	0.1	0.1	0.1	0.1	0.1	−0.1	−0.1	0.1	0.1	0.1	−0.1	0.0	−0.1	−0.1	0.1	0.1	0.0	0.1	0.1
*Oil-a-17-1*	1.0	0.1	0.1	0.1	−0.1	−0.1	0.1	−0.1	0.1	0.1	0.1	0.1	0.1	0.1	0.1	0.1	0.1	−0.1	−0.1	−0.1	−0.1
*Oil-a-18-1*	2.1	−0.3	−0.3	−0.3	−0.3	−0.3	−0.3	−0.3	−0.3	−0.3	−0.3	−0.3	−0.3	−0.3	−0.3	−0.3	−0.3	−0.3	−0.3	−0.3	−0.3
*Oil-a-18-2*	7.2	−0.2	−0.2	−0.2	−0.2	0.2	−0.2	−0.2	−0.2	−0.2	−0.2	−0.2	−0.2	−0.2	−0.2	−0.2	−0.2	0.3	0.3	0.2	0.3
*Oil-a-18-3*	0.9	−0.2	0.2	−0.2	−0.2	−0.2	−0.2	−0.2	−0.2	−0.2	−0.2	−0.2	−0.2	−0.2	−0.2	−0.2	0.2	−0.2	−0.2	0.2	0.2
*Oil-a-18-4*	1.2	0.3	0.3	0.3	0.3	−0.3	0.3	0.3	0.3	0.3	0.3	0.3	0.3	0.3	0.3	0.3	0.3	0.3	0.3	0.3	0.3
*Oil-a-20-1*	0.3	−0.2	−0.2	−0.2	−0.2	−0.2	−0.2	−0.2	−0.2	−0.2	−0.2	−0.2	−0.2	−0.2	−0.2	−0.2	−0.2	0.2	0.2	−0.2	−0.2
*Oil-a-20-2*	0.3	0.4	0.4	0.4	0.4	0.4	0.4	0.4	0.4	0.4	0.4	0.4	0.4	0.4	0.4	0.4	0.4	0.4	0.4	0.4	0.4
*Oil-a-20-3*	10.6	0.0	0.0	0.2	−0.2	−0.2	0.0	0.2	0.0	0.2	0.2	0.0	0.1	0.1	0.1	0.1	−0.2	0.0	0.0	0.2	0.2
*Oil-a-20-4*	0.3	−0.2	−0.2	−0.2	−0.2	−0.2	−0.2	−0.2	−0.2	−0.2	−0.2	−0.2	0.2	0.2	0.2	0.2	−0.2	−0.2	−0.2	−0.2	−0.2
*Oil-a-20-5*	0.3	0.5	−0.5	0.5	0.5	0.5	−0.5	0.5	0.5	0.5	0.5	0.5	0.5	0.5	0.5	0.5	0.5	0.5	0.5	0.5	0.5
*Oil-a-20-6*	2.2	0.3	0.3	0.3	0.3	0.3	−0.3	0.3	−0.3	0.3	0.3	0.3	0.3	0.3	0.3	0.3	0.3	0.3	0.3	0.3	0.3
*Oil-a-20-7*	8.8	−0.9	−0.1	0.1	0.1	−0.9	0.1	0.2	0.1	0.1	0.1	0.2	0.2	0.1	0.1	0.1	0.1	0.5	0.1	0.5	0.5
*Oil-a-20-8*	1.5	−0.1	−0.1	−0.1	−0.1	−0.1	0.1	−0.1	0.1	−0.1	−0.1	0.1	0.1	0.1	0.1	−0.1	0.1	0.1	0.1	0.1	0.1
*Oil-a-20-9*	0.4	−0.4	−0.4	−0.4	−0.4	−0.4	−0.4	−0.4	−0.4	−0.4	−0.4	−0.4	−0.4	−0.4	−0.4	−0.4	−0.4	−0.4	−0.4	0.4	0.4
*Oil-a-20-10*	0.3	0.3	0.3	0.3	0.3	0.3	0.3	0.3	0.3	0.3	0.3	0.3	0.3	0.3	0.3	0.3	0.3	0.3	0.3	0.3	0.3
Oil content (%)		15.8	17.1	17.2	17.4	17.4	17.6	17.7	17.7	17.8	17.8	22.8	22.9	22.9	22.9	23.0	23.0	23.0	23.5	23.7	24.1
Positive allele no.		20	25	24	19	23	24	24	22	23	23	33	29	29	27	27	29	29	30	31	31
Negative allele no.		30	25	26	31	27	26	26	28	27	27	17	21	21	23	23	21	21	20	19	19

### Genetic Differentiation Among Ecoregion Subpopulations in the CSLRP

The AMOVA showed that significant genetic differentiations existed among ecoregions as well as among landraces within each ecoregion for seed oil traits in the CSLRP (Supplementary Table 9). The within ecoregion variance component was much greater than the among ecoregion variance component, which implied that a great variation happened in each ecoregion after the ancient ancestors moved to different ecoregions. The whole QTL-allele matrix was then separated into six ecoregion matrices for each trait. The independence of the allele frequency distribution of detected QTL among the ecoregions was tested with Chi-square criterion, and 28, 56, and 30 QTLs showed significant differentiation among the ecoregions for SOC, OAC, and LAC, respectively (Supplementary Table 6).

It was assumed that allele with the highest frequency was the original allele of one locus while allele with lowest frequency was newly happened or mutant allele. There are six kinds of differentiation patterns (Table 6): (1) As *Oil-a-03-1* and *Oil-a-11-1*, the first major allele was positive while negative allele was its mutant, multiple alleles existed on a locus and all the alleles disseminated to all six ecoregions or commonly shared by all ecoregions. (2) As *Oil-a-15-2*, the first major allele was negative while positive allele was its mutant, multiple alleles existed at a locus and all the alleles disseminated to all six ecoregions. (3) As *Oil-a-03-2*, only two alleles existed on this locus, one was dominant, the other was newly happened but disseminated to other ecoregions, the major allele was negative while positive was its mutant. (4) The opposite situation to the third pattern with the major allele was positive. (5) As *Oil-a-18-2* and *Oil-a-20-3*, the first major allele was negative while positive allele was its mutant, the three major alleles disseminated across all the ecoregions, while another three alleles, as newly happened, only disseminated in some ecoregions. (6) As *Oil-a-20-7*, the opposite situation to the fifth pattern with the first major allele was positive.

**Table 6:** Allele frequency distribution for large effect (*R*^2^ > 3%) seed oil content QTL in CSLRP.

**QTL**	***R^2^***	**χ^2^*p*-value**	**Allele**	**Effect**	**Frequency**	**Ecoregion frequency**					
						**I**	**II**	**III**	**IV**	**V**	**VI**
*Oil-a-03-1*	4.58	<0.001	1	0.18	0.49	0.34	0.52	0.56	0.43	0.57	0.56
			2	−0.36	0.35	0.32	0.21	0.38	0.48	0.30	0.41
			3	0.18	0.16	0.34	0.28	0.07	0.10	0.14	0.03
*Oil-a-03-2*	3.29	0.082	A	−0.29	0.93	0.89	0.98	0.93	0.94	0.97	0.85
			G	0.29	0.07	0.11	0.02	0.07	0.06	0.03	0.15
*Oil-a-11-1*	3.06	<0.001	1	0.05	0.55	0.53	0.25	0.69	0.73	0.59	0.51
			2	0.33	0.19	0.15	0.22	0.17	0.15	0.24	0.26
			3	0.04	0.16	0.26	0.26	0.07	0.10	0.11	0.18
			4	−0.43	0.10	0.06	0.26	0.07	0.02	0.05	0.05
*Oil-a-15-2*	4.25	0.009	1	−0.25	0.60	0.51	0.67	0.58	0.65	0.54	0.49
			2	0.11	0.24	0.28	0.17	0.17	0.21	0.41	0.41
			3	0.14	0.16	0.21	0.16	0.25	0.13	0.05	0.10
*Oil-a-18-2*	7.17	<0.001	1	−0.22	0.41	0.43	0.63	0.47	0.32	0.16	0.21
			2	−0.19	0.31	0.19	0.15	0.33	0.46	0.32	0.38
			3	−0.39	0.16	0.15	0.08	0.08	0.15	0.27	0.36
			4	0.22	0.07	0.09	0.10	0.07	0.02	0.08	0.03
			5	0.27	0.04	0.09	0	0.03	0.02	0.14	0
			6	0.26	0.02	0.04	0.01	0.01	0.01	0.03	0
			7	0.05	0.01	0.02	0.02	0	0	0	0.03
*Oil-a-20-3*	10.60	0.075	1	−0.22	0.45	0.40	0.43	0.43	0.45	0.49	0.51
			2	0.17	0.23	0.30	0.23	0.22	0.23	0.19	0.18
			3	0.06	0.19	0.11	0.23	0.19	0.17	0.27	0.21
			4	0.04	0.07	0.13	0.05	0.08	0.07	0.05	0
			5	−0.04	0.04	0	0.07	0.04	0.07	0	0
			6	0.00	0.03	0.06	0	0.03	0.01	0	0.10
*Oil-a-20-7*	8.82	<0.001	1	0.24	0.42	0.36	0.44	0.38	0.46	0.22	0.62
			2	0.14	0.39	0.26	0.43	0.43	0.33	0.73	0.23
			3	0.55	0.06	0.13	0.06	0.04	0.06	0.05	0.03
			4	0.05	0.06	0.11	0.02	0.07	0.08	0	0.05
			5	−0.08	0.04	0.04	0.01	0.07	0.04	0	0.08
			6	−0.90	0.03	0.11	0.05	0.01	0.02	0	0

*The allele frequency distribution among the six ecoregions for all detected QTL of the seed oil traits in CSLRP was listed in Supplementary Table 6*.

Based on the individual QTL differentiation among ecoregions, ecoregion matrices differentiated accordingly. The obvious differentiation appeared in the existence of specific ecoregion alleles. More than 63% alleles in each trait were found in all six ecoregions while some alleles (<5% for each trait) existed in two or even one ecoregion(s) which were considered as specific ecoregion alleles (Supplementary Figure 4, Supplementary Tables 6, 10). Among the six ecoregions, ecoregion I, and ecoregion II had more ecoregion specific alleles than other ecoregions, for example, allele “7” of Gm04_BLOCK96_9670209_9789832 existed only in ecoregion I. Recognizing ecoregion-specific alleles is of great importance in studying allele evolution and ecoregion-allele relationships.

### Genomic Selection for Optimal Crosses in Recombination Breeding

Based on the detected QTL-allele matrices, the recombination potentials of all possible single crosses were estimated using with- and without-linkage prediction model, and more recombination cycles is needed for realization of the without-linkage prediction in breeding programs (Table 7). There was no large difference in predicted values between the two models, and therefore results from with-linkage prediction were mentioned in the present study. The maximum value within ecoregions for SOC and OAC could be achieved as 24.20% (ecoregion II) and 38.38% (ecoregion I), while the maximum value among different ecoregions for SOC and OAC could be achieved as 24.76% [N05283.2 (III) × N05193 (II)] and 40.30% [N23538 (I) × N23561 (II)], indicating that the crosses with parents from different ecoregions could achieve higher transgressive value for SOC and OAC. However, the minimum value within ecoregions for LAC was 2.37% [N24278 (I) × N23538 (I)], which was even less than the value among the different ecoregions. From the prediction results, 15 potential crosses within and among ecoregions for each trait were recommended for seed oil traits breeding programs (Supplementary Table 11). These predictions indicate a great potential of seed oil traits improvement through recombination breeding in CSRLP based on the genetic dissection of population using RTM-GWAS.

**Table 7:** The predicted seed oil content, oleic acid, and linolenic acid content for all possible single crosses.

**Trait**	**Ecoregion**	**Extreme phenotype**	**Cross number**	**Predicted phenotype**		
				**Mean**	**Min**.	**Max**.
Seed oil	I	24.14	1,081	21.38 (21.40)	19.51 (19.51)	23.73 (23.58)
	II	22.98	3,741	21.53 (21.57)	19.20 (19.20)	24.20 (24.61)
	III	23.67	2,556	21.17 (21.19)	19.46 (19.46)	23.41 (23.34)
	IV	22.33	3,486	20.89 (20.91)	19.20 (19.20)	22.33 (22.39)
	V	22.50	666	21.22 (21.22)	19.87 (19.92)	23.66 (23.64)
	VI	21.34	741	20.95 (20.97)	18.18 (18.18)	22.94 (23.13)
	Entire	24.14	66,795	21.22 (21.24)	18.18 (18.18)	24.76 (24.97)
Oleic acid	I	37.09	1,081	26.63 (27.59)	19.17 (19.68)	38.38 (40.29)
	II	31.06	3,741	24.39 (25.00)	18.60 (18.67)	34.02 (36.09)
	III	31.25	2,556	23.46 (24.00)	16.98 (16.98)	33.33 (33.38)
	IV	34.49	3,486	23.77 (24.46)	17.63 (17.72)	31.21 (32.72)
	V	27.07	666	23.96 (24.40)	20.04 (20.26)	28.74 (28.66)
	VI	30.87	741	23.33 (24.09)	17.83 (17.84)	29.14 (31.29)
	Entire	37.09	66,795	24.26 (24.93)	16.24 (16.24)	40.30 (41.44)
Linolenic acid	I	2.86	1,081	6.00 (5.88)	2.37 (2.46)	8.79 (8.65)
	II	5.00	3,741	6.61 (6.49)	3.81 (3.94)	8.68 (8.68)
	III	5.35	2,556	6.96 (6.82)	5.20 (4.81)	9.15 (9.15)
	IV	4.31	3,486	7.06 (6.90)	4.79 (4.51)	9.29 (9.15)
	V	6.62	666	7.23 (7.12)	5.84 (5.84)	8.57 (8.57)
	VI	6.53	741	7.10 (7.03)	5.82 (5.72)	9.04 (9.04)
	Entire	2.86	66,795	6.79 (6.66)	2.37 (2.46)	9.29 (9.24)

*Extreme phenotype values were the maximum (seed oil and oleic acid content) and minimum (linolenic acid content) phenotype values observed in the ecoregion. The numbers outside parentheses were the predicted values based on linkage model, while in parentheses were based on independent assortment model*.

The optimal crosses for comprehensive improvement of SOC, OAC, and LAC were also estimated based on the prediction (Table 8). This was picked up from individual results of each trait. Since the individual trait prediction was based on linkage model, the comprehensive prediction should also be considered to include linkage information. Among the top 20 comprehensive high seed oil trait crosses, the best ones were from crosses between an ecoregion I parent (such as N23679 and N23538) and an ecoregion II parent (such as N09445 and N05193). It seems that the best seed oil trait parental materials are mainly located in ecoregion I with several materials from other ecoregions.

**Table 8:** The predicted optimal crosses for seed oil traits improvement.

**Parent 1**					**Parent 2**					**Cross**		
**Accession**	**Eco**.	**SOC**	**OAC**	**LAC**	**Accession**	**Eco**.	**SOC**	**OAC**	**LAC**	**SOC**	**OAC**	**LAC**
N23538	I	21.66	37.09	3.08	N24274	I	24.14	22.12	6.63	23.19 (22.99)	34.76 (36.48)	3.43 (3.52)
N23679	I	21.46	35.11	2.86	N23583	II	19.79	12.26	9.72	23.08 (23.19)	31.69 (31.87)	4.32 (4.39)
N23679	I	21.46	35.11	2.86	N21277	II	18.72	16.18	9.09	23.27 (23.55)	33.45 (33.40)	3.89 (3.98)
N23679	I	21.46	35.11	2.86	N09445	II	19.74	18.73	8.33	23.40 (23.84)	34.44 (34.64)	3.44 (3.61)
N23679	I	21.46	35.11	2.86	N07378.1	II	18.66	16.77	9.17	23.18 (23.54)	33.55 (33.36)	3.86 (3.86)
N23679	I	21.46	35.11	2.86	N05193	II	21.22	18.08	8.08	23.97 (24.32)	34.86 (34.37)	3.02 (3.17)
N23538	I	21.66	37.09	3.08	N23583	II	19.79	12.26	9.72	23.03 (23.16)	33.77 (33.66)	3.82 (3.87)
N23538	I	21.66	37.09	3.08	N21277	II	18.72	16.18	9.09	23.12 (23.44)	35.21 (34.79)	3.35 (3.40)
N23538	I	21.66	37.09	3.08	N09445	II	19.74	18.73	8.33	23.27 (23.88)	36.68 (36.73)	2.94 (3.01)
N23538	I	21.66	37.09	3.08	N07378.1	II	18.66	16.77	9.17	23.13 (23.41)	35.22 (34.66)	3.44 (3.41)
N23538	I	21.66	37.09	3.08	N05193	II	21.22	18.08	8.08	24.09 (24.36)	36.30 (35.93)	2.58 (2.74)
N24281	I	17.82	25.49	7.30	N05193	II	21.22	18.08	8.08	23.13 (23.36)	30.84 (30.68)	4.39 (4.16)
N23679	I	21.46	35.11	2.86	N03328	V	22.51	16.93	8.16	23.49 (23.57)	32.34 (32.29)	4.27 (4.23)
N23538	I	21.66	37.09	3.08	N03328	V	22.51	16.93	8.16	23.37 (23.56)	34.17 (33.99)	3.71 (3.83)
N05193	II	21.22	18.08	8.08	N08983.1	I	18.59	29.14	6.05	23.56 (23.83)	30.94 (30.48)	3.96 (4.10)
N24605	II	19.06	27.08	6.47	N05193	II	21.22	18.08	8.08	23.28 (23.49)	30.38 (30.73)	4.11 (4.22)
N23561	II	20.76	31.06	5.00	N05193	II	21.22	18.08	8.08	23.35 (23.64)	31.75 (33.66)	3.81 (3.94)
N05193	II	21.22	18.08	8.08	N00134	III	20.13	31.13	5.51	23.46 (23.84)	30.51 (30.45)	4.35 (4.14)
N05283.2	III	23.67	20.78	6.90	N23538	I	21.66	37.09	3.08	23.09 (23.05)	34.44 (35.49)	3.41 (3.60)
N01434	III	20.78	31.25	5.35	N05193	II	21.22	18.08	8.08	23.43 (23.69)	30.50 (30.85)	4.35 (4.22)

*Eco., ecoregion. SOC, seed oil content; OAC, oleic acid content; LAC, linolenic acid content. The numbers outside parentheses were the predicted values based on linkage model, while in parentheses were based on independent assortment model*.

### The Candidate Genes That Confer the Seed Oil Traits in the CSLRP

**Figure 3:**
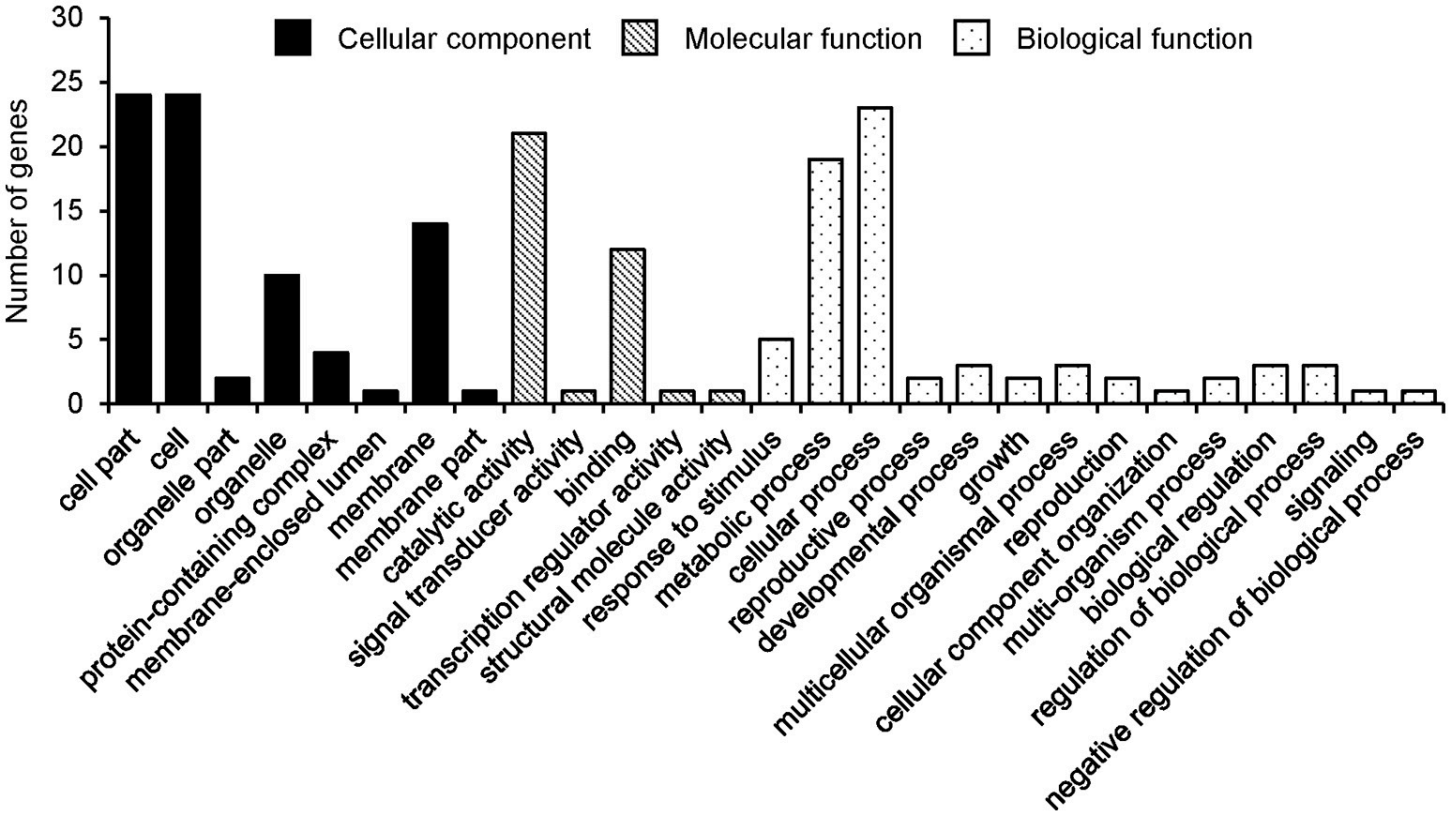
Gene ontology classification of annotated genes for seed oil content.

From the detected 13, 19, and 12 major QTLs of the three seed oil traits, a total of 38, 27 and 25 genes were annotated for SOC, OAC, and LAC, respectively (Supplementary Tables 12–14). These candidate genes, 28, 15, and 15 genes were selected according to chi-square test and grouped into three GO categories, i.e., biological process, cellular component, and molecular function for SOC, OAC, and LAC, respectively (Figure 3, Supplementary Figure 5).

There were two major common QTLs between OAC and LAC, and no common QTL was found for SOC (Supplementary Table 3). The SNPLDB Gm04_BLOCK96_9670209_9789832 was detected to be associated with OAC (*Oleic-a-04-2*) and LAC (*Linolenic–a-04-2*), which explained the highest phenotypic variation for both OAC (13.20%) and LAC (16.83%). In the marker region, *Glyma04g11250* (Haloacid dehalogenase-like hydrolase superfamily protein) and *Glyma04g11330* (Transducin/WD40 repeat-like superfamily protein) were identified as candidate genes. The SNPLDB Gm18_BLOCK530_61189911_61190505 was detected to be associated with OAC (*Oleic-a-18-9*) and LAC (*Linolenic–a-18-4*), which explained a high portion of phenotypic variation for both OAC (4.61%) and LAC (2.79%). Six candidate genes were identified within this region, including *Glyma18g52590* [delta(3), delta(2)-enoyl CoA isomerase 1] which was previously reported to be essential for the beta-oxidation of unsaturated fatty acids (Geisbrecht et al., 1998).

In the present gene systems of three seed oil traits, there were eight genes on two loci shared by OAC and LAC with major contribution. It seemed that more shared genes appear between the two fatty acid traits rather than with SOC. However, SOC as a super trait should have a more genetic relationship with its fatty acid components theoretically, and therefore further studies on SOC are needed.

## Discussion

### The Advantages of the Innovative RTM-GWAS Procedure

Firstly, RTM-GWAS is based on multi-allelic SNPLDB marker rather than bi-allelic SNP marker used by other GWAS methods. The SNPLDB marker can match genetic locus with varied number of alleles, and detecting QTL by SNPLDB marker should be more effective and powerful than bi-allelic SNP marker for germplasm populations. Secondly, RTM-GWAS is based on an efficient two-stage association analysis, where the markers were preselected by single-locus model followed by multi-locus model stepwise regression. In multi-locus model analysis, loci are jointly fitted and tested in a joint linear model, and the experiment-wise error is controlled under the normal significance level. Therefore, no additional multiple correction is needed. This is different from GWAS methods based on single-locus model where a large number of independent statistical inferences are considered and the experiment-wise testing has to be completed through correction, such as Bonferroni correction, while the correction in fact is not a model test but an arbitrary adjustment. Since QTL detection is carried out at the second stage under multi-locus model in RTM-GWAS, the total variation explained by detected QTL will not overflow trait heritability.

Although there are already GWAS methods that implement multi-locus model, such as MLMM (Segura et al., 2013) and mrMLM (Wang et al., 2016), they are designed for bi-allelic SNP marker only and are not applicable to the multi-allelic SNPLDB marker. However, the mrMLM method based on SNP marker was also performed and 12, 25, and 9 SNPs in a total of 46 SNPs were detected with 37.16, 51.83, and 36.06% phenotype variation contribution for SOC, OAC, and LAC, respectively (Supplementary Table 15). In comparison with mrMLM, 13, 19, and 12 SNPLDBs in a total of 42 SNPLDBs were detected with 55.38, 67.47, and 56.63% of phenotype variation contribution using RTM-GWAS under the same significance level of 0.0002 (Supplementary Table 3). Among the 46 SNPs, 7 SNPs with 1.09–3.76% phenotype variation contribution were found to overlap with 7 large contribution (*R*^2^ > 1%) QTLs identified by RTM-GWAS. RTM-GWAS detected the large effect loci detected by mrMLM, but it should be noted that mrMLM results were based on SNP marker while RTM-GWAS results were based on SNPLDB marker, and it might be not an exact comparison since multiple SNPs included in a SNPLDB which may provide more allele information than a single SNP. In fact, each GWAS method has its own advantages and disadvantages and fits respective purposes. That is why researchers try to improve it from different aspects. For example, some researchers aimed to find a handful of quantitative trait nucleotides (QTN) for identifying some major genes. As a quantitative trait, especially such as oil content which is the final product of a series of biological processes, is conferred by many genes or QTLs, what we concerned is how to identify the genetic system of a trait in a germplasm population, and this is different from targeting on a few loci for individual gene study. Unfortunately, our results have not reached the goal yet due to the difficulty in controlling the experimental error and there is still a relatively small part of the genetic variation to be explained.

### The Detected QTL Systems of Seed Oil Traits in Comparison With Those in the Literature

The QTLs in SoyBase (http://soybase.org) were incorporated into 54, 25, and 21 conformity QTLs according to their position and precision (Supplementary Table 1). These conformity QTLs can match 37, 19, and 9 QTLs detected in this study, therefore, about 13, 79, and 29 QTLs detected in the present study for SOC, OAC, and LAC are newly discovered, respectively (Tables 3, 4). Here the QTLs of SOC in SoyBase were obtained from 30 different mapping populations (mainly recombinant inbred lines) with 49 different parental materials, those of the OAC in SoyBase were from 9 different populations with 17 different parental accessions, and those of the LAC in SoyBase were from 10 different populations with 20 different parental accessions. However, those are only making comparisons and supports, not necessarily a direct strong validation, because the previous results are not necessarily complete and exact. As the previous QTL studies on seed oil traits are still quite limited, logically to compare back with the previous results is only a check and not enough as a validation. In this case, to evaluate the present results, what we considered is how much improvement had been made for the new procedure and what the new finds of the study were. The number of overlapped QTLs is not necessary to reflect the reliability and efficiency of a new method or a new study. Obviously, the present study provided more QTL-allele information than previous linkage mapping results with more precision and less expense. One reason is that materials used in the present study are a gene reservoir of the Chinese landrace population as from which the soybean origin area covering a wide range of genetic variance, and another reason is the high efficiency of RTM-GWAS procedure which provided the detected QTLs with 82.52–90.29% contribution to the phenotypic variation.

### Population Characterization Based on QTL-Allele Matrices

Based on the relatively thorough detection of QTL-alleles through RTM-GWAS procedure, the QTL-allele matrix has provided a new tool for characterizing the populations. From the QTL-allele matrix, all kinds of genetic parameters can be obtained, such as QTL number, allele number, allele frequency, allele effect, genetic diversity, etc. The genetic differentiation among populations can be detected based on individual QTL, a group of QTLs, and a subpopulation. From the changes of allele frequencies, the evolutionary relationship among alleles can be detected. If the QTL-allele matrix is linked to ecological conditions, eco-genetics knowledge can be further revealed. In the present study, we have tried to conduct the analysis, but further results are to be explored. We believe the genome-wide QTL-allele matrix may be an important tool in population genetic studies.

### Approaches to Achieve Genomic Selection for Transgressive Seed Oil Traits in the CSLRP

The genetic structure in term of QTL-allele matrix of the CSLRP showed that both positive and negative alleles existed in each accession for the seed oil traits, denoting a great recombination potential. According to prediction based on the linkage model, the SOC and OAC can be achieved as high as 24.76 and 40.30% and the LAC can be achieved as low as 2.37%. In plant breeding, this is the first stage of genomic selection, selection for optimal cross. The selected crosses are potential for next genomic selection stage, selection for best progenies to realize the potential or to obtain progenies with 24.76% SOC. There are some difficulties associated with classic genomic selection based on GEBVs (Meuwissen et al., 2001) in plant breeding, i.e., the large number of segregating progenies involving extremely high genotyping cost and uncertainty of marker-breeding value relationship due to the black box procedure. According to the present study, all genome-wide 50, 98, and 50 QTLs along with their 136, 283, and 154 alleles rather than all genome-wide SNPs of progenies should be genotyped for SOC, OAC, and LAC improvement, respectively. In such case, a small marker chip can meet the requirements of genomic selection for superior progenies, or other high throughput molecular marker technologies, such as high throughput PCR, is to be further explored. Therefore, the suggested novel GS procedure based on QTL-allele matrix in plants composes of GS for optimal crosses and GS for progeny selection. The prerequisite of this novel GS strategy is the precise and thorough QTL-allele dissection in the gene reservoir.

The above example of genomic selection for optimal crosses involves only three seed oil traits (Table 8). Since plant breeding usually involves multiple traits, genomic selection for comprehensive optimal crosses can be conducted by using multiple matrices or a weighted combination of multiple matrices. Similarly, GS for progenies incorporating multiple traits can be achieved using multiple trait markers or a weighted combination of multiple trait markers.

In addition, the allele effects obtained from the RTM-GWAS were additive effects since the materials used were inbred landraces. The additive by additive interaction effect was not considered in RTM-GWAS, but it was usually not large according to the reported linkage mapping results, especially for the three oil traits. Therefore, we recommend the prediction results should be relatively relevant to breeding programs.

### Candidate Major QTL/Genes of Seed Oil Traits for Further Study

In addition to using the whole QTL-allele information in genomic selection for breeding, the information revealed through major QTL/genes can be further studied for exploring the gene network of the traits. The two QTLs located on chromosome Gm04, and Gm18 along with their annotated genes are important for further study as mentioned in the above text. Furthermore, chromosome Gm20 with four major SOC QTLs explaining up to 24.07% of the phenotypic variation was the most important chromosome for SOC, on which a number of studies also reported some SOC QTL using linkage mapping and association mapping. But no major QTL was found on chromosome Gm20 for both OAC and LAC. Chromosome Gm04 was found to be important for OAC and LAC, explaining up to 19.54 and 21.28% of the phenotypic variation, respectively, but no major QTL was found for SOC. These results suggested that seed oil content and fatty acid content may be determined by different QTLs on different chromosomes. Therefore, a great potential exists for recombination between seed oil content and fatty acid content in the CSRLP.

The expressional level of the candidate genes and the pathway of the candidate genes were analyzed, but only six QTL/genes were found to be related to OAC and LAC. Gene expression analysis showed that *Glyma10g01590* (*Oleic-a-10-1*), *Glyma11g30090* (*Oleic-a-11-4*), *Glyma13g23800* (*Oleic-a-13-4*), *Glyma17g34450* (*Oleic-a-17-5*), and *Glyma17g34510* (*Oleic-a-17-5*) were showed high expressional levels at oil accumulation stage of seed development. Pathway analysis showed that only *Glyma09g17170* (*Linolenic-a-09-1*) was fatty acid metabolism gene. The results indicated that oil content and fatty acid content were complex traits, and the existing information is relatively limited, further studies are needed to find major seed oil QTL/genes. In addition, GWAS results of a trait in germplasm population are obtained from one-direction inference, and validation of the results finally depends on finding all the genes through experimental molecular biology. Since our results can detect many more QTLs/genes in comparison to other GWAS procedures with only a handful QTLs detected, it seems difficult to completely validate all the QTLs through gene cloning in a short period. More effort is needed to explore the gene system from the QTL system of the three seed oil traits.

## Author Contributions

JG, YZ, and JH conceived and designed the experiments. YZ, HW, SM, JZ, and GX performed the field experiments. JH, YL, SY, JZ, and TZ performed the genome sequencing. YZ and JH analyzed and interpreted the results. YZ, JH, and JG drafted the manuscript and all authors contributed to the manuscript revision.

### Conflict of Interest Statement

The authors declare that the research was conducted in the absence of any commercial or financial relationships that could be construed as a potential conflict of interest.

## Supplementary Material

Supplementary Figure 1 Manhattan plots of genome-wide association studies of three seed oil traits using MLM method. **(A)** Seed oil content; **(B)** oleic acid content; **(C)** linolenic acid content. The blue horizontal line represents significance level of 0.05, and the red horizontal line represents Bonferroni-adjusted significance level of 0.05.

Supplementary Figure 2 The information of oleic acid QTL-allele matrix. **(A)** Effect distribution of 283 alleles on the 98 loci for the oleic acid content (%). **(B)** Distribution of number of alleles on the 98 loci for oleic acid content (%). **(C)** The graphical presentation of oleic acid QTL-allele matrix. The horizontal axis represents accessions arranged in a rising order of oleic acid content (%), while the vertical axis represents QTL arranged in a rising order of their positive allele frequency. Each row represents the allele distribution among accessions for a QTL, while each column indicates the allele constitution of an accession over all QTLs. Allele effects are expressed in color cells with warm colors indicating positive effects and cool colors indicating negative effects, and the color depth indicates effect size.

Supplementary Figure 3 The information of linolenic acid QTL-allele matrix. **(A)** Effect distribution of 154 alleles on the 50 loci for the linolenic acid content (%). **(B)** Distribution of the number of alleles on the 50 loci for linolenic acid content (%). **(C)** The graphical presentation of linolenic acid QTL-allele matrix. The horizontal axis represents accessions arranged in a rising order of linolenic acid content (%), while the vertical axis represents QTL arranged in a rising order of their positive allele frequency. Each row represents the allele distribution among accessions for a QTL, while each column indicates the allele constitution of an accession over all QTLs. Allele effects are expressed in color cells with warm colors indicating positive effects and cool colors indicating negative effects, and the color depth indicates effect size.

Supplementary Figure 4 The number of alleles shared among different number of ecoregions.

Supplementary Figure 5 Gene ontology classifications of annotated genes for seed **(A)** oleic acid and **(B)** linolenic acid content.

Supplementary Table 1 Reported QTLs of three seed oil traits in SoyBase (http://soybase.org).

Supplementary Table 2 Joint analysis of variance of three seed oil traits in CSLRP.

Supplementary Table 3 The detected SNPLDBs associated with three seed oil traits under a significance level of 0.0002 in CSLRP.

Supplementary Table 4 The detected small effect (*R*^2^ < 0.01) SNPLDBs associated with oleic acid and linolenic acid content in CSLRP.

Supplementary Table 5 Distribution of detected QTL of three seed oil traits in CSLRP.

Supplementary Table 6 Allele frequency distribution among six ecoregions for the detected QTL of three seed oil traits in CSLRP.

Supplementary Table 7 QTL-allele constitution of twenty accessions with the lowest and highest oleic acid content (%) for the detected oleic acid QTLs in CSLRP.

Supplementary Table 8 QTL-allele constitution of twenty accessions with the lowest and highest linolenic acid content (%) for the detected linolenic acid QTLs in CSLRP.

Supplementary Table 9 Analysis of molecular variance among six ecoregions of CSLRP based on the SNPLDBs associated with three seed oil traits.

Supplementary Table 10 The ecoregion-specific alleles on the QTLs of three seed oil traits.

Supplementary Table 11 The predicted optimal crosses of the seed oil traits within and among ecoregions.

Supplementary Table 12 The candidate genes annotated from the detected SNPLDBs of seed oil content in CSLRP.

Supplementary Table 13 The candidate genes annotated from the detected SNPLDBs of oleic acid content in CSLRP.

Supplementary Table 14 The candidate genes annotated from the detected SNPLDBs of linolenic acid content in CSLRP.

Supplementary Table 15 The detected SNPs associated with seed oil traits using mrMLM in CSLRP.
