# Supplementary figures and images for: Detecting the QTL-Allele System of Seed Oil Traits Using Multi-Locus Genome-Wide Association Analysis for Population Characterization and Optimal Cross Prediction in Soybean
Source: Front Plant Sci. 2018 Dec 5;9:1793. doi: 10.3389/fpls.2018.01793 (PMC6290252; doi:10.3389/fpls.2018.01793)

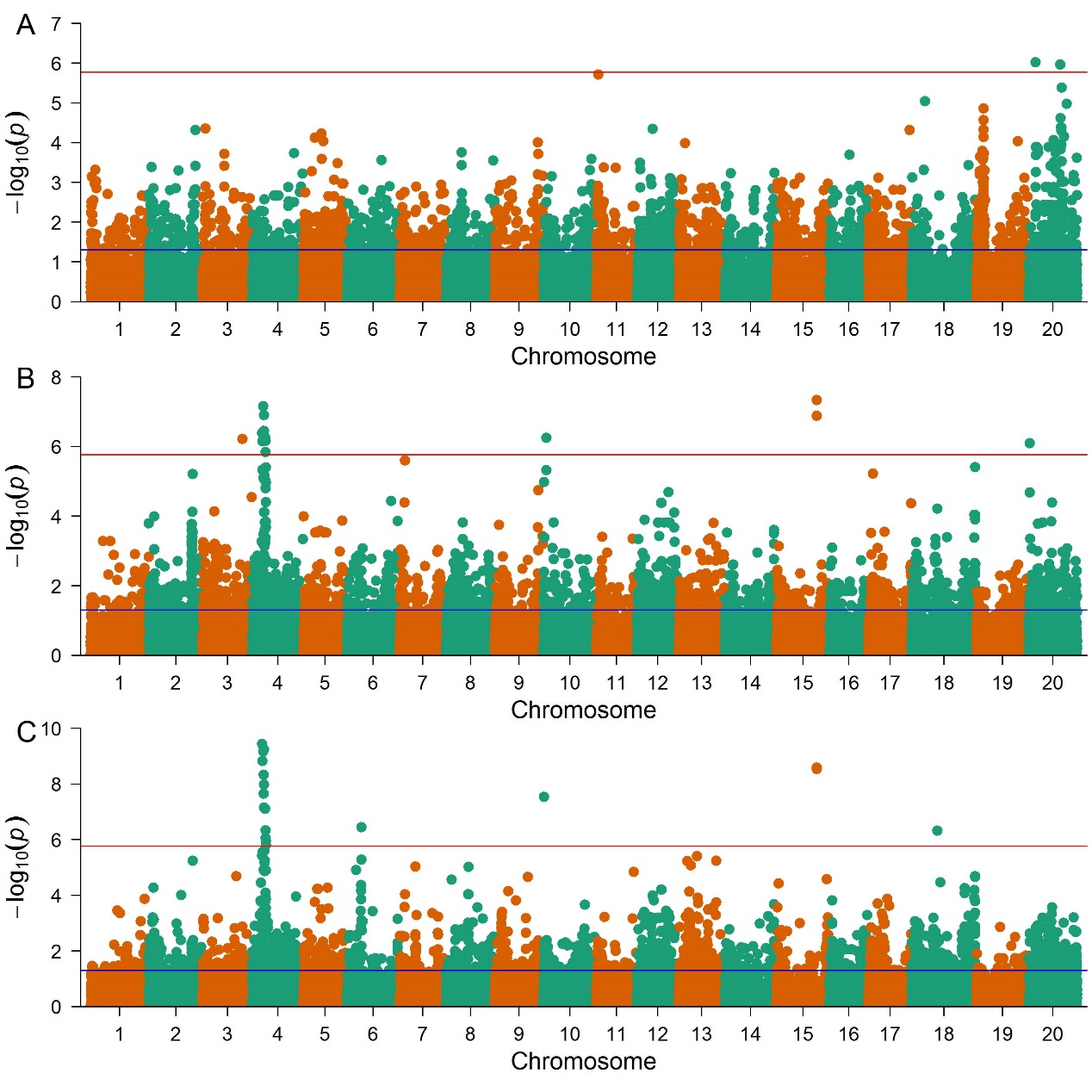

Supplement: Supplementary Figure 1 — Manhattan plots of genome-wide association studies of three seed oil traits using MLM method. (A) Seed oil content; (B) oleic acid content; (C) linolenic acid content. The blue horizontal line represents significance level of 0.05, and the red horizontal line represents Bonferroni-adjusted significance level of 0.05. [file Image_1.TIF]

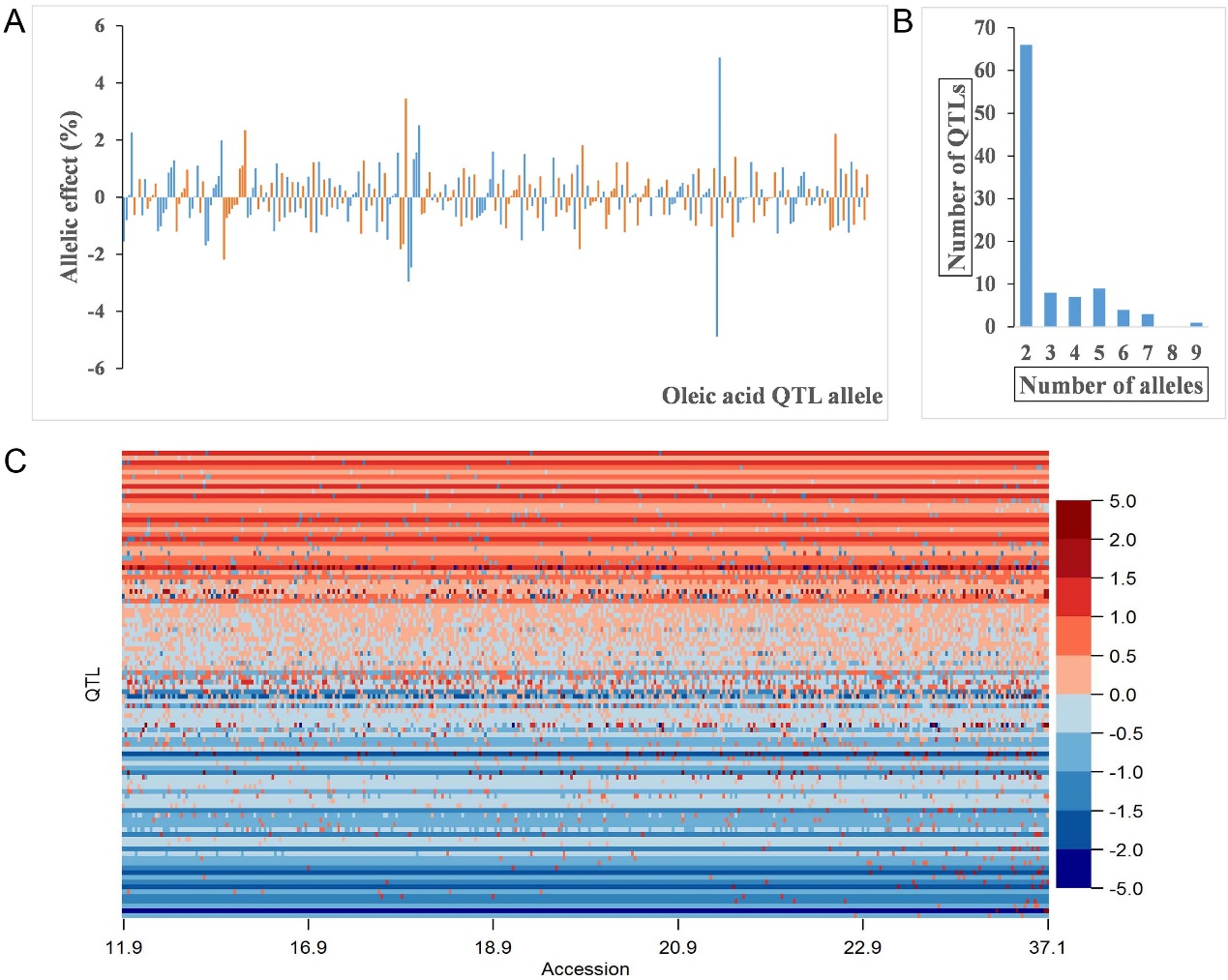

Supplement: Supplementary Figure 2 — The information of oleic acid QTL-allele matrix. (A) Effect distribution of 283 alleles on the 98 loci for the oleic acid content (%). (B) Distribution of number of alleles on the 98 loci for oleic acid content (%). (C) The graphical presentation of oleic acid QTL-allele matrix. The horizontal axis represents accessions arranged in a rising order of oleic acid content (%), while the vertical axis represents QTL arranged in a rising order of their positive allele frequency. Each row represents the allele distribution among accessions for a QTL, while each column indicates the allele constitution of an accession over all QTLs. Allele effects are expressed in color cells with warm colors indicating positive effects and cool colors indicating negative effects, and the color depth indicates effect size. [file Image_2.TIF]

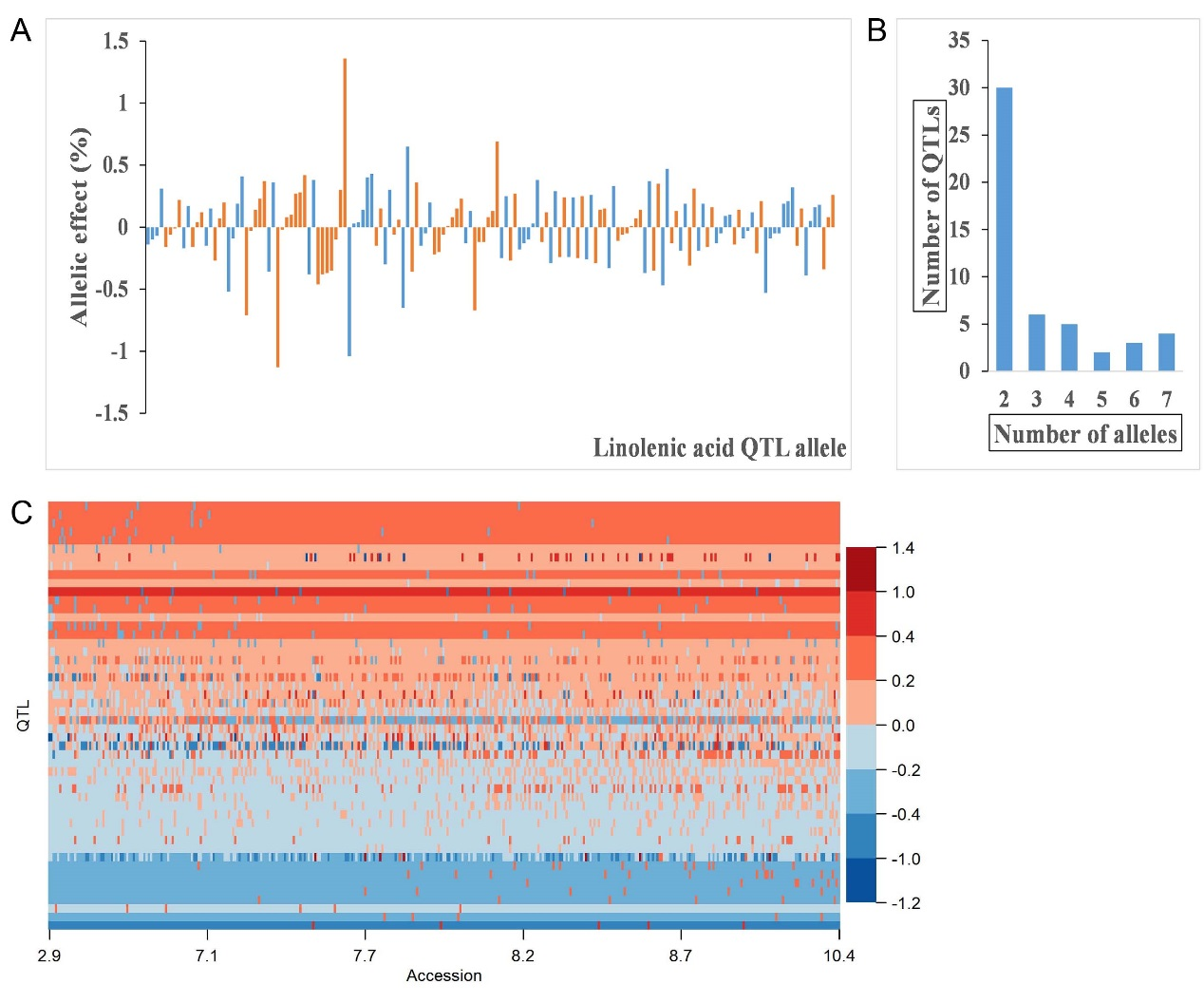

Supplement: Supplementary Figure 3 — The information of linolenic acid QTL-allele matrix. (A) Effect distribution of 154 alleles on the 50 loci for the linolenic acid content (%). (B) Distribution of the number of alleles on the 50 loci for linolenic acid content (%). (C) The graphical presentation of linolenic acid QTL-allele matrix. The horizontal axis represents accessions arranged in a rising order of linolenic acid content (%), while the vertical axis represents QTL arranged in a rising order of their positive allele frequency. Each row represents the allele distribution among accessions for a QTL, while each column indicates the allele constitution of an accession over all QTLs. Allele effects are expressed in color cells with warm colors indicating positive effects and cool colors indicating negative effects, and the color depth indicates effect size. [file Image_3.TIF]

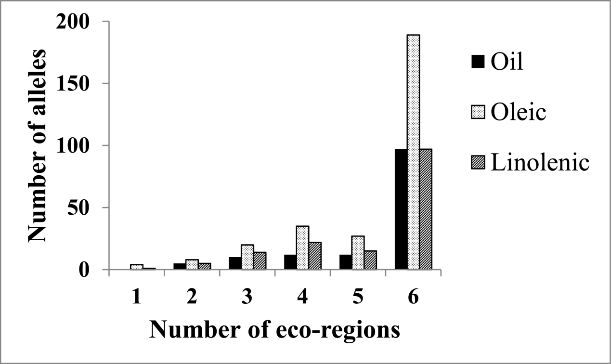

Supplement: Supplementary Figure 4 — The number of alleles shared among different number of ecoregions. [file Image_4.TIF]

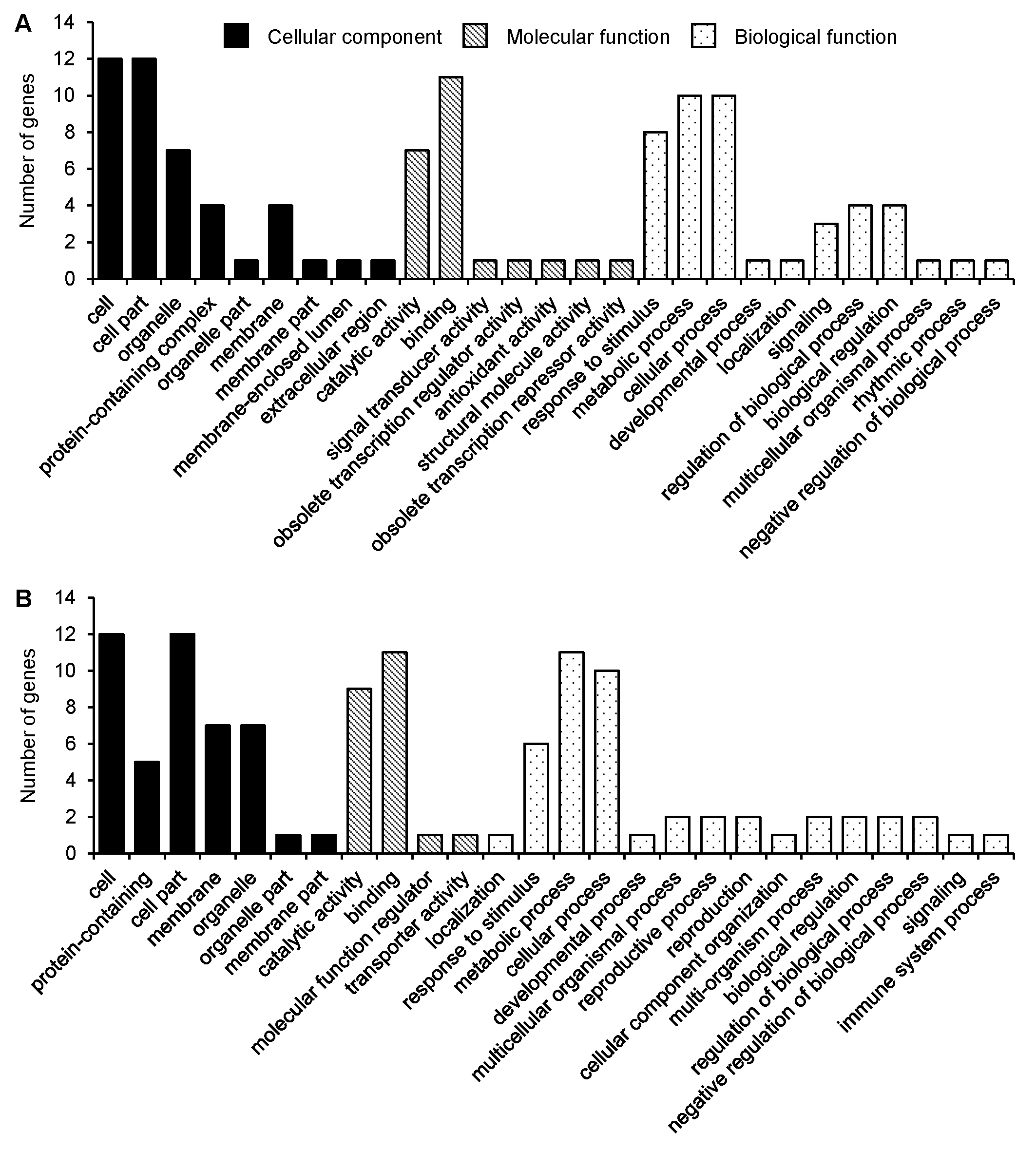

Supplement: Supplementary Figure 5 — Gene ontology classifications of annotated genes for seed (A) oleic acid and (B) linolenic acid content. [file Image_5.TIFF]
